# An *App* knock-in rat model for Alzheimer’s disease exhibiting Aβ and tau pathologies, neuronal death and cognitive impairments

**DOI:** 10.1038/s41422-021-00582-x

**Published:** 2021-11-17

**Authors:** Keliang Pang, Richeng Jiang, Wei Zhang, Zhengyi Yang, Lin-Lin Li, Makoto Shimozawa, Simone Tambaro, Johanna Mayer, Baogui Zhang, Man Li, Jiesi Wang, Hang Liu, Ailing Yang, Xi Chen, Jiazheng Liu, Bengt Winblad, Hua Han, Tianzi Jiang, Weiwen Wang, Per Nilsson, Wei Guo, Bai Lu

**Affiliations:** 1 School of Pharmaceutical Sciences, IDG/McGovern Institute for Brain Research, Tsinghua University-Peking University Joint Center for Life Sciences, Tsinghua University, Beijing, China; 2 R&D Center for the Diagnosis and Treatment of Major Brain Diseases, Research Institute of Tsinghua University in Shenzhen, Shenzhen, Guangdong China; 3 Beijing Tiantan Hospital, Advanced Innovation Center for Human Brain Protection, Capital Medical University, Beijing, China; 4 Department of Neurobiology, Care Sciences and Society, Division of Neurogeriatrics, Center for Alzheimer Research, Karolinska Institutet, Stockholm, Sweden; 5 Department of Otorhinolaryngology Head and Neck Surgery, The First Hospital of Jilin University, Changchun, China; 6 CAS Key Laboratory of Mental Health, Institute of Psychology, Chinese Academy of Sciences, and Department of Psychology, University of Chinese Academy of Sciences, Beijing, China; 7 Brainnetome Center, Institute of Automation, Chinese Academy of Sciences, Beijing, China; 8 Research Center for Brain-inspired Intelligence, National Laboratory of Pattern Recognition, Institute of Automation, School of Future Technology, University of CAS, and CAS Center for Excellence in Brain Science and Intelligence Technology, Chinese Academy of Sciences, Shanghai, China; 9 Theme Aging, Karolinska University Hospital, Huddinge, Sweden

**Keywords:** Biological techniques, Ageing

## Abstract

A major obstacle in Alzheimer’s disease (AD) research is the lack of predictive and translatable animal models that reflect disease progression and drug efficacy. Transgenic mice overexpressing amyloid precursor protein (*App*) gene manifest non-physiological and ectopic expression of APP and its fragments in the brain, which is not observed in AD patients. The *App* knock-in mice circumvented some of these problems, but they do not exhibit tau pathology and neuronal death. We have generated a rat model, with three familiar *App* mutations and humanized Aβ sequence knocked into the rat *App* gene. Without altering the levels of full-length APP and other APP fragments, this model exhibits pathologies and disease progression resembling those in human patients: deposit of Aβ plaques in relevant brain regions, microglia activation and gliosis, progressive synaptic degeneration and AD-relevant cognitive deficits. Interestingly, we have observed tau pathology, neuronal apoptosis and necroptosis and brain atrophy, phenotypes rarely seen in other APP models. This *App* knock-in rat model may serve as a useful tool for AD research, identifying new drug targets and biomarkers, and testing therapeutics.

## Introduction

Alzheimer’s disease (AD) is one of the most prominent age-related diseases and the major cause of elderly disability, with progressive impairments in memory, personality, and other cognitive functions. AD has brought the heaviest social burden to the modern aging society.^1,2^ The pathological hallmarks for AD include amyloid β peptide (Aβ)-containing senile plaques, tau-containing neurofibrillary tangles, progressive neuronal death, and neuroinflammation.^2^ As such, amyloid and tau cascade hypotheses have been proposed to explain AD pathogenesis.^3–5^ Despite the progress in understanding some of the pathogenic mechanisms, efforts in developing disease-modifying therapies for AD have so far been unsuccessful.^6,7^ Many factors may have contributed to this but one of the major hurdles appears to be the shortage of animal models that fully recapitulate the disease pathogenesis and therefore are useful for testing experimental drugs.^8–11^ Over decades hundreds of models have been developed, but few could genuinely reproduce the major neuropathologic phenotypes seen in AD patients.^9–11^ This may have contributed to the fact that quite a few candidate AD drugs shown to be effective in AD animal models have failed in clinical trials.^11^ The majority of AD models are transgenic mice overexpressing the human *App* gene. The most widely used are the PDAPP and Tg2576 mice.^12,13^ which overexpress *App* gene with Indiana and Swedish mutation respectively. Some mouse models carry both APP and PSEN1 mutations such as APPswe/PS1_M46L_, APPswe/PSEN1dE9 and 5× FAD mice.^14–16^ However, these animal models exhibit little neuronal death, no tau pathology, and develop Aβ pathology in ectopic brain areas not seen in human AD brains.^9,11^ It remains uncertain whether the synaptic and behavioral deficits seen in these animals were due to Aβ or other peptidergic products derived from non-physiological expression and processing of APP.^17,18^ The overexpression or mis-expression of mutated APP and/or PSEN1 in transgenic animals with ‘non-self’ promotors have led to many problems as well as unphysiological phenotypes, which have been discussed extensively.^11,18,19^ Further, nearly in 99% of the transgenic models the sites of transgene insertion have not been mapped and therefore the observed phenotypes cannot be entirely attributed to the biology of the transgene.^20^

Knock-in technology may circumvent some of these problems. Mice with targeted knock-in of single London mutation in the *App* gene displayed only mild cognitive impairment without obvious AD pathology.^21^ When two or three familial AD mutations were introduced into mice, typical AD phenotypes including Aβ plaques, neuroinflammation (gliosis), synaptic loss and cognitive impairment have been observed.^19^ However, two critical features are lacking in this model: the tau pathology and neuronal death/brain atrophy.^18,19^ These shortcomings may hamper its utility in basic research and drug development. Given the differences in brain size, cerebrospinal fluid (CSF) volume, and most importantly genomic structures and sequences between mouse and human, modeling AD in mouse has not been ideal. For decades, attempts have been made to develop other animal models that are physiologically, genetically and morphologically closer to humans.^9^

Compared to mice, genetic manipulation in rats has been less advanced due to shortages of tools.^22,23^ However, rats are more resemblance to human in physiology and behaviors than mice. Their larger body size makes it easier for surgical manipulations and for collecting blood/CSF samples.^23^ More importantly, the genomic similarity between rat and human, such as the alternative splicing of *tau* gene, suggests that it might be more appropriate to model AD in rats.^24^ Several transgenic rat models, such as McGill-R-Thy1-APP,^25^ TgF344-AD,^26^ and APP + PS1,^27^ were generated, and some showed aspects of AD pathologies,^9,28,29^ including neuroinflammation,^30,31^ synaptic loss,^32,33^ and cognitive impairments.^34,35^ In particular, tau pathology has been reported in TgF344-AD rats at 16 months but not 6 months.^26^ Cell death was detected in the subiculum of McGill-R-Thy1-APP rats,^36^ and hippocampus and cerebral cortex of TgF344-AD rats after 16 months of age.^26^ Interestingly, transgenic rat models for other neurodegenerative diseases such as Parkinson’s disease seem to exhibit a more accurate representation of the human disease compared to transgenic mice bearing the same transgene.^37,38^ However, these models suffer the same shortcomings of transgenic technology: non-physiological phenotypes due to overexpression of the transgene. Recently, knock-in technology has been applied to modeling AD in rat. Unfortunately, these rat models, which are with single *App* mutation leading to little Aβ deposits, do not exhibit significant AD pathologies.^39–42^

Accordingly, it is highly desirable to develop alternative models that can faithfully recapitulate majority of the pathogenic mechanisms in AD using knock-in technology. In this study, we have generated an *App* knock-in rat line harboring Swedish-Beyreuther/Iberian-Arctic mutations using the CRISPR/Cas9 technology. This rat model exhibits a comprehensive set of AD-relevant pathological, cellular and behavioral phenotypes rarely seen in other APP models. It may serve as a useful tool in aiding both AD research and drug discovery.

## Results

### Generation of the *App* knock-in rats

**Fig. 1:**
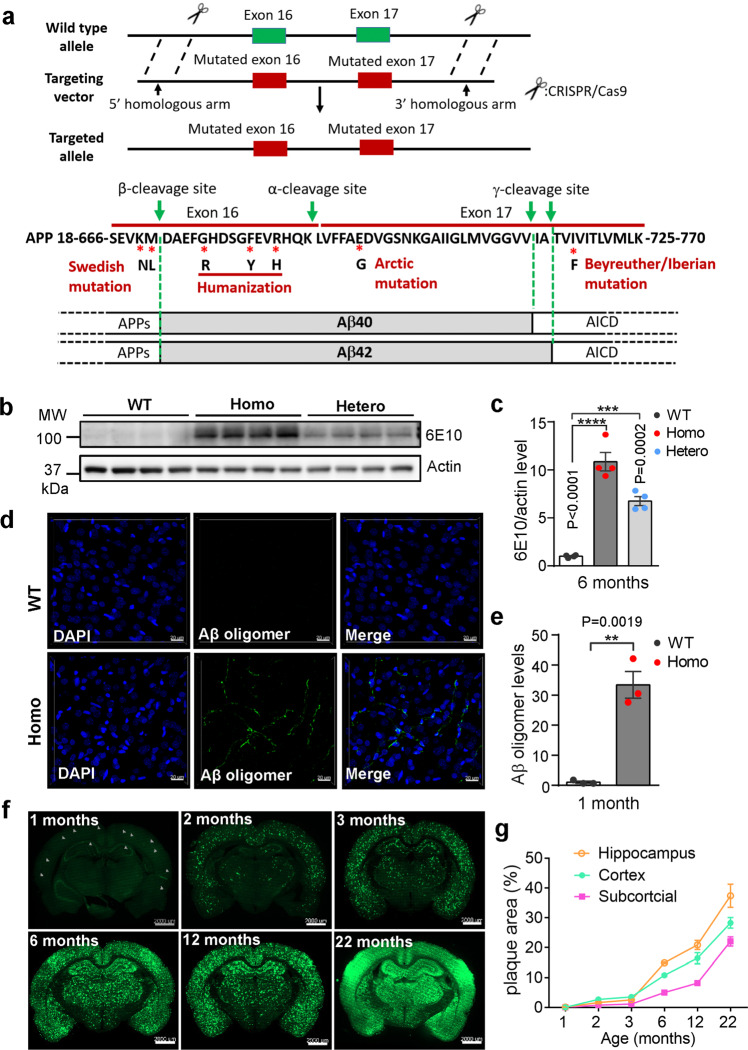
Aβ pathology in *App*^*NL-G-F*^ rat brains. **a** Gene-editing strategy to generate knock-in rat with three familial *App* mutations, using CRISPR-Cas9 technologies. Upper: App wild-type allele, targeting vector and targeted allele. The donor molecule comprises a 5′ homologous arm upstream of exon 16, and a 3′ homologous arm downstream of exon 17. Lower: Diagram showing all the important sites on the APP sequence. Green arrows: cleavage sites by 3 secretases. *: key mutation sites identified by human genetics and 3 humanized amino acids G676R, F681Y, and R684H. **b**, **c** Expression of full-length amyloid precursor protein (FL-APP). FL-APP containing humanized Aβ sequence was detected by Western blotting using antibody 6E10 (specific for human Aβ sequence) in 6-month-old wild type (WT), *App*^*NL-G-F/NL-G-F*^ (Homo) and *App*^*NL-G-F/WT*^ (Hetero) rats. Representative Western blot is shown in **b** and quantification of FL-APP expression by densitometry in **c**. *n* = 4 animals in each genotype. Data in this and all bar graphs in other figures are presented as means ± SEM. Statistical analyses were carried out using one-way ANOVA. *P* values are shown in the bar graphs. **d**, **e** Formation of Aβ oligomers. Representative microphotographs from 1-month-old WT and Homo rat brain sections are shown in **d**, and quantification of fluorescence is shown in **e**. (*n* = 3–4 rats, and at least 3 sections from each rat were used for quantifications. Unless stated otherwise, statistical analyses in this and all other figures were carried out using student’s *t*-test, *P* values are shown in the bar graphs, Scale bars, 20 μm.) **f** Aβ deposition in *App*^*NL-G-F*^ rat brains. Brain sections from different ages of homozygous *App*^*NL-G-F*^ rats were stained with an antibody specific for Aβ plaques and images were captured with a light-sheet fluorescence microscope. Arrows in the “1-month” figure indicate representative plaques. **g** Quantification of the plaque areas for different brain regions is shown in **f**. *n* = 3 rats for each time point, Scale bars, 2000 μm.

The genomic DNA sequences of rat and human *App* gene were searched from NCBI database, and the sequences for Aβ42 peptide, encoded by the sequence of exon 16, and 17 of *App* gene, were analyzed and compared. To construct the *App* knock-in rats, the sequence for Aβ42 and the surrounding mutation sites of rat *App* were substituted by human sequences. Swedish double mutations (K670N substitution and M671L substitution) were introduced into exon 16, and Beyreuther/Iberian (I716F substitution) and Arctic (E693G substitution) mutations were introduced into exon 17. Furthermore, the Aβ sequence was humanized by introducing mutations leading to the substitutions G676R, F681Y, and R684H. Figure 1a provides a scheme indicating the mutations introduced to obtain the modified rat *App* using CRISPR/Cas9 technology. After testing the efficiency, the CRISPR/Cas9 components were injected into the Sprague Dawley (SD) rat zygotes. Approximately 21–23 days after the transplantation, the F0 rats carrying the desired chimeric *App* gene were born and crossed with wild type SD rats to obtain F1 rats. The scheme of the gene editing and probing strategy is illustrated in Supplementary information, Fig. S1a. Southern blotting was performed with 5’ probe and 3’ probe to verify the correct recombination in F1 rats (Supplementary information, Fig. S1b). The genotypes of the F1 rats were also determined using PCR and DNA sequencing for further confirmation of mutations and humanized sequences. The homozygous *App* knock-in (named hereafter homozygous *App*^*NL-G-F*^) rats were obtained by crossing over F1 rats. A common concern of CRISPR-Cas9 based technologies is the risk of off-target activity.^43,44^ To investigate the potential off-target sites in these knock-in rats, we used CCTop-CRISPR/Cas9 target online predictor^45^ and selected sgRNA sequences. The selected sgRNA sequences produced no off-target modifications in any functional regions of the genome (data not shown). In addition, we examined the body weight and core body temperature of *App*^*NL-G-F*^ rats. Compared to WT rats, *App*^*NL-G-F*^ rats showed a small reduction in body weight but no difference in core body temperature (Supplementary information Fig. S1c, d). Similar observation of weight loss was also reported in AD patients and mouse models.^46–48^

### Aβ pathology in *App*^*NL-G-F*^ rats

While Aβ pathology could be found in most of the previously generated *App* transgenic models, overexpression of APP raises concerns on whether the phenotypes seen in these animals are the results of APP mutation or overexpression, or both.^18^ To examine the expression level of chimeric APP with mutations in *App*^*NL-G-F*^ rats, an antibody (6E10) specifically recognizing the human Aβ (1–16) sequence was used. An allelic dose-dependent expression of chimeric APP was detectable in the hippocampus of homozygous (*App*^*NL-G-F/NL-G-F*^*)* and heterozygous *(App*^*NL-G-F/WT*^) rats compared to wild type (WT or *App*^*WT/WT*^) rats (Fig. 1b, c), whereas the protein levels of full-length APP as measured by the N-terminal antibody 22C11, as well as its various proteolytic fragments (such as CTF-α and CTF-β, detected by the C-terminal antibody A8717) were comparable in WT and homozygous *App*^*NL-G--F*^ rats (Supplementary information, Fig. S1e–i). Thus, unlike APP overexpressing transgenic mouse models, the newly generated knock-in rat model, *App*^*NL-G-F*^, retains the endogenous levels of APP and its metabolites.

Aβ oligomerization is a critical intermediate state for the formation of Aβ plaques.^49^ Immunofluorescent staining using Aβ oligomer-specific monoclonal antibody (OMAB) detected Aβ oligomer pathology in as early as 1-month-old, male homozygous *App*^*NL-G-F*^ rats, but no Aβ oligomers were observed in the WT counterpart (Fig. 1d, e). Substantial amount of Aβ oligomers was also detected in 3-month-old heterozygous *App*^*NL-G-F*^ rats (Supplementary information, Fig. S2a). One of the hallmarks of AD is the presence of Aβ plaques in the brain. Using an antibody that specifically reacts with Aβ plaques, we detected traces of Aβ deposition as early as 1-month postnatal in the homozygous (Fig. 1f) and 4-month in heterozygous, male rats (Supplementary information, Fig. S2b). Aβ deposition in homozygous rats is approximately three times faster than that in the heterozygous and increases in an age-dependent manner (Fig. 1g; Supplementary information, Fig. S2b). Notably, the Aβ plaques were rarely seen in the cerebellum even in the 10-month-old homozygous *App*^*NL-G-F*^ rats (Supplementary information, Fig. S2c). We also examined Aβ plaques in female homozygous rats. Aβ deposition in female also exhibits an age-dependent increase. Interestingly, it appears that in the three brain areas examined, Aβ deposits progress more rapidly in females than males, especially after 6-month of age (Supplementary information, Fig. S2d, e). The presence of abundant Aβ oligomers, plaques, their spatiotemporal distribution, and lack of Aβ deposit in the cerebellum in *App*^*NL-G-F*^ rats broadly resemble the amyloid pathology observed in human AD brains.

### Tau pathology in *App*^*NL-G-F*^ rats

**Fig. 2:**
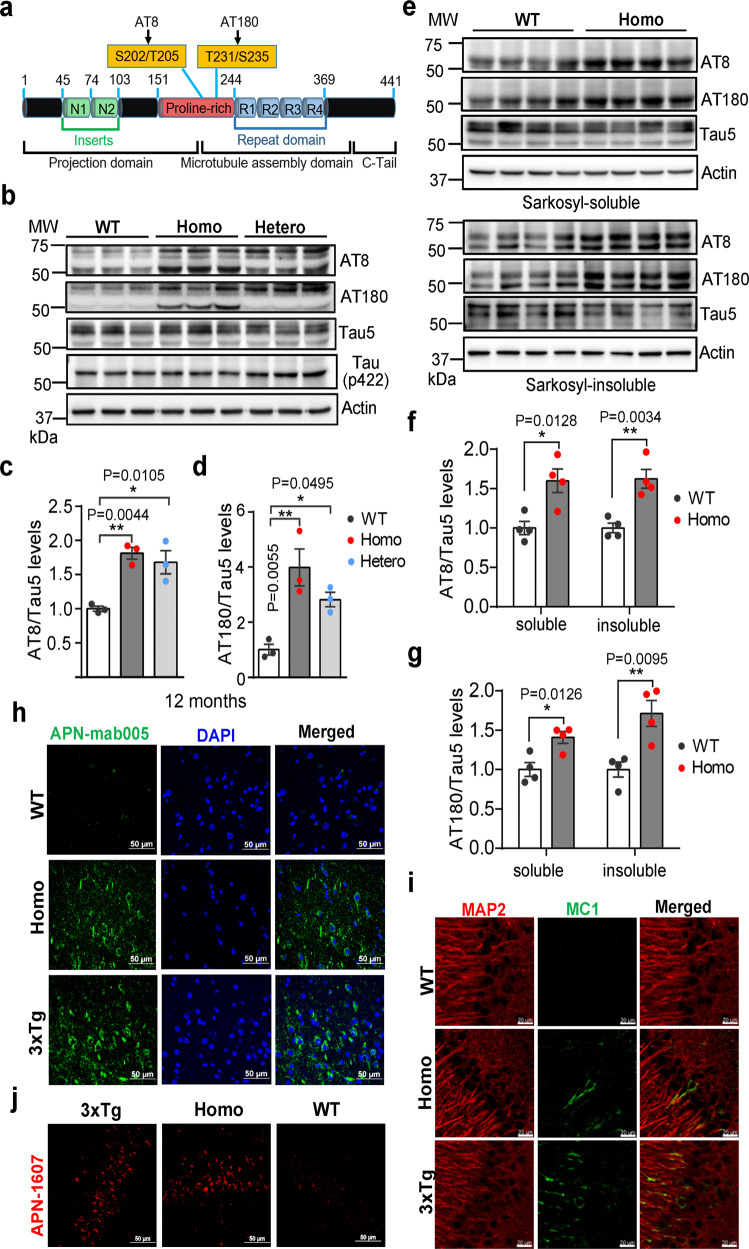
Tau pathology in *App*^*NL-G-F*^ rat brains. **a** A diagram of human tau protein structure showing the major phosphorylation sites. The six major isoforms of tau in the rat brains are distinguished by the presence or absence of N-terminal inserts (N1, N2) (green) or R2 domain (blue). Two representative phosphorylation sites can be identified by the phospho-specific antibody AT8 and AT180 (indicated by arrows). **b**–**d** Tau phosphorylation in *App*^*NL-G-F*^ rats. RIPA fractions of cortical lysates from 12-month-old rats were immunoblotted with antibodies recognizing phosphorylated tau at residue S202/T205 (AT8), T231 (AT180), or S422, and total tau protein (Tau5) was also detected as the control. The upper and lower bands in the AT8 and AT180 blots represent different tau isoforms (**b**). The levels of tau phosphorylation, quantified by densitometry and expressed as AT8/Tau5 and AT180/Tau5, are shown in **c** and **d**. *n* = 3, Statistics: one-way ANOVA. **e**–**g** Tau phosphorylation in the sarkosyl-soluble or insoluble fractions of *App*^*NL-G-F*^ rat brain lysates. The different fractions of cortical lysates from 12-month-old rats were immunoblotted with AT8, AT180, Tau5 or actin antibodies. The upper and lower panels represent the immunoblotting of sarkosyl-soluble or insoluble fractions respectively (**e**). The levels of tau phosphorylation, quantified by densitometry and expressed as AT8/Tau5 and AT180/Tau5, were shown below (**f**, **g**). *n* = 4. **h** Detection of tau aggregation using APN-mab005, a specific antibody for aggregated tau. Frozen brain sections from 12-month-old male 3× Tg-AD mice, 12-month-old male WT and homozygous *App*^*NL-G-F*^ rats were double stained with APNmab005 and DAPI in cortical region. Scale, 50 μm. **i** Detection of disease-specific conformational change of tau using MC1 antibody. Brain sections from 12-month-old male 3× Tg-AD mice, 22-month-old WT and homozygous *App*^*NL-G-F*^ rats were double stained with the MAP2 and MC1 antibodies in hippocampal CA1 region. Scale, 20 μm. **j** Detection of tau aggregation using APN-1607, a fluorescent dye specific for aggregated tau. Brain sections from 12-month-old male 3× Tg-AD mice, 22-month-old WT and homozygous *App*^*NL-G-F*^ rats were stained with APN-1607 in hippocampal CA1 region. Scale, 50 μm.

Abnormally hyper-phosphorylated tau protein is the major component of neurofibrillary tangles (NFT), another key pathology of AD. Studies on human postmortem tissues indicated that the phosphorylation level of tau isolated from autopsied AD brain is 3- to 4-fold higher than that of non-AD brains.^50^ To date, more than 45 phosphorylation sites have been identified in tau protein isolated from AD brains.^51^ However, no pathologic tau aggregation including NFTs have been reported in any previously published *App* transgenic or knock-in mouse models.^9^ Similar to humans, rats contain 6 isoforms of tau (3 × 4R, 3 × 3R), although the ratio of 4R/3R tau isoforms is different (9:1 in rats; 1:1 in humans). Comparatively, mice express only three 4R isoforms of tau, but lack 3R tau.^24^ To examine the tau pathology in *App*^*NL-G-F*^ rats, the RIPA (containing SDS), sarkosyl-soluble or sarkosyl-insoluble fractions from cortex lysates were immunoblotted for phosphorylated tau at residues S202/T205 (detected by AT8 antibody), T231 (by AT180 antibody), S422 and total tau protein (Fig. 2a, b, e). In the RIPA (containing SDS) fractions, there was almost a 2-fold increase in the phosphorylation of the Thr231and Ser202 of tau protein in the 12-month-old *App*^*NL-G-F*^ rats (Fig. 2b–d, the entire blots were shown in Supplementary information, Fig. S3j), but no significant changes were identified at S422 epitope. We also examined tau phosphorylation in the 3-, 6- and 9-month-old homozygous *App*^*NL-G-F*^ rats. The level of AT8/Tau increased in both 6- and 9-month but not in 3-month-old homozygous rats, whereas that of AT180-labeled tau5 increased only in 9- but not in 3- or 6-month-old *App*^*NL-G-F*^ (Supplementary information, Fig. S3a–i). In the sarkosyl-soluble and sarkosyl-insoluble fractions, phosphorylation of Thr231and Ser202 of tau was also increased significantly in the 12-month-old homozygous *App*^*NL-G-F*^ rats. Consistent with what was previously reported in AD brains, more phosphorylated isoforms (bands in gel) of tau were present in the sarkosyl-insoluble fractions compared to the sarkosyl-soluble fractions (Fig. 2e–g).

Next, we used APN-mab005, a newly developed monoclonal antibody (an antibody drug that blocks tau aggregation and propagation^52^) that recognizes specifically aggregated tau, mostly tau species segregated into the synaptic/membrane compartments in synaptosomes prepared from AD brains. APN-mab005 detected immunoreactivity only in cortical neurons in 12-month-old homozygous *App*^*NL-G-F*^ rats, but not WT control rats (Fig. 2h). In contrast, APN-mab005 detected few if any immunoreactivities in the hippocampus of 12-month-old APP-PS1 and 5× FAD mice, two commonly used AD mouse models (Supplementary information, Fig. S3k). As a positive control, 12-month-old 3× Tg-AD mice, which overexpress the mutant tau protein MAPT-P301L, exhibited similar APN-mab005 immunofluorescence (Fig. 2h bottom). In the much older (22-month-old) *App*^*NL-G-F*^ rats, much stronger APN-mab005 positive immunoreactivities were detected, and the labeling seemed to be associated with cytosol and dendrites, but not nucleus (arrows in Supplementary information, Fig. S3l).

Conformationally altered tau, which could be detected by MC1 antibody in rat,^26^ is another critical intermediate state for pathological aggregation of tau protein seen only in AD but not healthy control brains.^53^ We observed MC1-positive immunofluorescence in MAP2 labeled hippocampus CA1 neurons in 22-month-old homozygous *App*^*NL-G-F*^ rats and 12-month-old 3× Tg-AD mice, but not WT control rats (Fig. 2i). We also used APN-1607 (also known as PM-PBB3), a PET tracer known to fluorescently label various aggregates tau species but not monomeric tau.^54,55^ We found significant fluorescence labeling in the CA1 region of 22-month-old *App*^*NL-G-F*^ rat brain, similar to that in 3× Tg-AD mice (positive control) (Fig. 2j). Further, we examined whether tau aggregates could lead to the formation of the NFT-like structures. Using the traditional method of thioflavin-S staining, positive signals were detected in 22-month-old *App*^*NL-G-F*^ rat brain sections (Supplementary information, Fig. S3m) and in 3× Tg-AD mice (positive control) but not in 12-month-old *App*^*NL-G-F*^ rat brain (data not shown). It should be pointed out that these signals did not exhibit typical NTF structures. In addition, we performed Bielschowsky silver staining and found some dark labeling that looked like dystrophic neurites in the entorhinal cortex of *APP* ^*NL-F-G*^ but not WT rats (22-month-old), although they did not exhibit typical tangle-like structures (data not shown).

Finally, we examined tau pathology in female homozygous rats. APN-mab005 and MC1-positive immunofluorescence were also observed in female *App*^*NL-G-F*^ rat brains at the age of 12-month. (Supplementary information, Fig. S4a, b). Taken together, these results demonstrated that knock-in of *App* mutations could lead to morphological and biochemical changes that resemble tau pathology in the *App*^*NL-G-F*^ rat model.

### Enhanced gliosis in *App*^*NL-G-F*^ rats

**Fig. 3:**
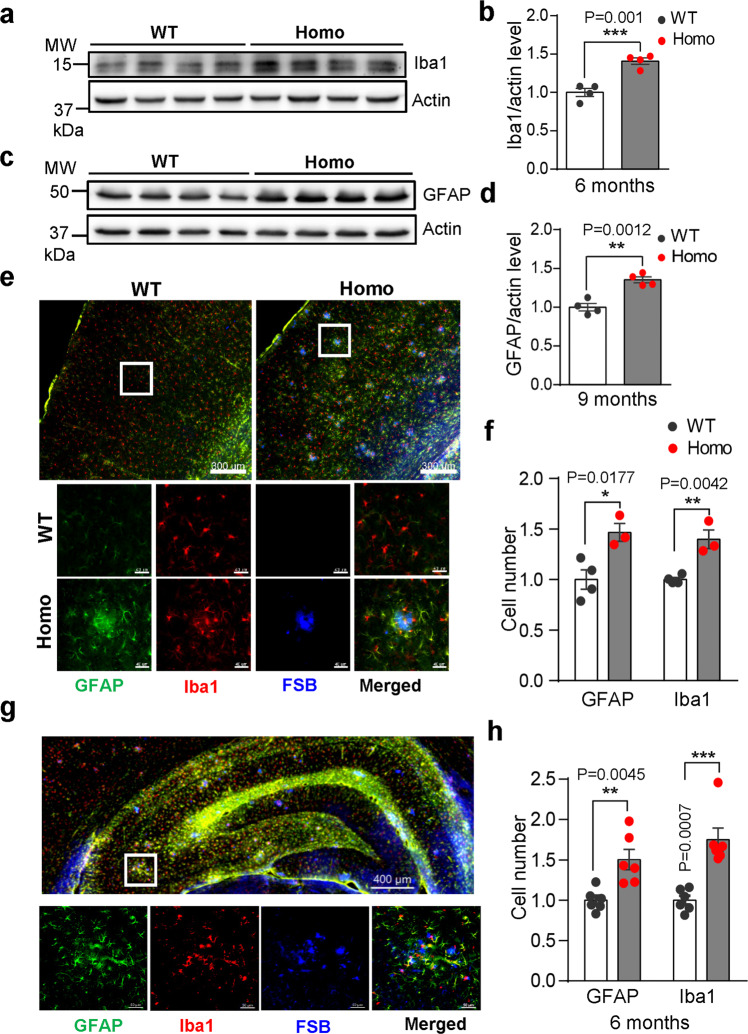
Enhanced gliosis in *App*^*NL-G-F*^ rat brains. **a**–**d** Microgliosis and astrocytosis in *App*^*NL-G-F*^ rats. Microglia marker Iba1 (**a**, **b**) or astrocyte marker GFAP (**c**, **d**) was detected in cortical lysates from 6-month-old WT and Homo rats using Western blotting. Representative immunoblots and quantification bar graphs are shown in the left (**a**, **c**) and right panels (**b**, **d**) respectively. *n* = 4 brains. **e**, **f** Representative microphotographs of microgliosis and astrocytosis in the cortex of *App*^*NL-G-F*^ rats. Gliosis were detected by triple staining of frozen sections from 6-month-old homozygous rat, using fluorostyryl benzene (FSB, for Aβ plaque), and GFAP (astrocytes) and Iba1 (microglia) antibodies. Scale, 300 μm. The boxed areas in the upper panels are shown in the panels below at a higher magnification (**e**). Scale bars, 40 μm. The numbers of astrocytes and microglia per sections are quantified and those of WT were normalized to 1 (**f**). *n* = 3 (Homo) or 4 (WT) rats. **g** Representative microphotographs of microgliosis and astrocytosis in the hippocampus of 6-month-old *App*^*NL-G-F*^ rats. The boxed areas in the upper panels are shown in the panels below at a higher magnification. **h** The numbers of astrocytes and microglia per sections are quantified and those of WT were normalized to 1. *n* = 6 rats.

Genetic epidemiological and experimental data all suggest a critical role of gliosis and neuroinflammation in AD.^56,57^ To study gliosis in *App*^*NL-G-F*^ rats, we first measured the protein levels of Iba1 (a microglia marker) and GFAP (an astrocyte marker) in *App*^*NL-G-F*^ rats. Western blot analyses revealed a significant increase in the levels of Iba1 and GFAP in the 6-month-old and 9-month-old *App*^*NL-G-F*^ rats respectively (Fig. 3a–d; Supplementary information, Fig. S5f), and both in the 12-month-old (Supplementary information, Fig. S5a–c), but not in the 3-month-old *App*^*NL-G-F*^ rats (Supplementary information, Fig. S5d, e). Note that there was also a small albeit significant increase in both Iba1 and GFAP in the 12-month-old heterozygous mutants (Supplementary information, Fig. S5a–c). We also examined morphological features of microglia and astrocyte activation. In addition to a marked increase of astrocytes and microglia in the cortical and hippocampal regions (and many other areas) of 6-month-old (Fig. 3e, g, upper panels, f, h), 12-, 22-month-old homozygous (Supplementary information, Fig. S5g), and 12-month-old heterozygous *App*^*NL-G-F*^ (Supplementary information, Fig. S5j), We also observed accumulation of microglia (red) and astrocytes (green) appeared to be highly concentrated around the Aβ plaques (blue, FSB) (Fig. 3e, g lower panels; Supplementary information, Fig. S5h). Quantification of immunofluorescence-positive cells revealed significant increases in the number of Iba1- and GFAP- cells (Fig. 3f, h; Supplementary information, Fig. S5i). The association of microgliosis and astrocytosis with Aβ plaques was confirmed by staining using an Aβ plaque-specific antibody (Supplementary information, Fig. S5k). The significant increase in glial cell numbers starting from 6-month in the mutant rats suggest that the gliosis was more likely to be due to an elevation of microglia and astrocytes proliferation, but not a decrease in glial cell death (overall cell death in the brain was increased, see below), nor the existence of more glia prior the disease onset (no difference in glia number in 3-month-old *App*^*NL-G-F*^ mice).

### Synaptic degeneration in *App*^*NL-G-F*^ rats

**Fig. 4:**
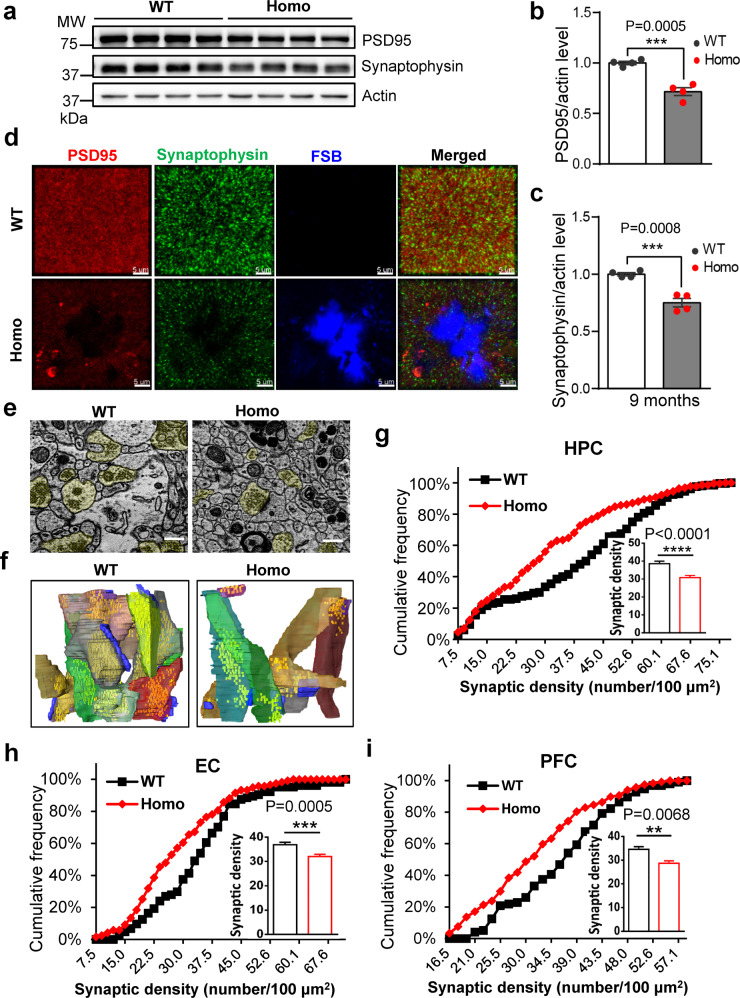
Synaptic degeneration in *App*^*NL-G-F*^ rats. **a**–**c** Levels of synaptic proteins in *App*^*NL-G-F*^ rats. Synaptosomal extracts from 9-month-old WT and Homo rat hippocampus were immunoblotted for presynaptic (synaptophysin) and postsynaptic (PSD-95) markers (**a**). Quantifications of synaptic protein levels are shown in the right (**b**, **c**). *n* = 4. **d** Representative immunostainings of synaptophysin and PSD-95. Brain sections from 9-month-old rats were triple immunostained with FSB for Aβ plaques, synaptophysin and PSD95 antibodies for presynaptic and postsynaptic markers, respectively. Note that in homozygous brain, there is a dramatic reduction in synaptic markers. Scale, 5 μm. **e**–**i** Quantitative EM analysis of synaptic loss in *App*^*NL-G-F*^ rats. **e** Representative EM sections showing synapses of hippocampus (HPC) from 6-month-old WT or Homo rat brains. The pre-synaptic elements are labeled in yellow. Scale, 500 nm. **f** 3D reconstruction of synapses from WT or homozygous rat brain. Presynaptic terminals in pseudo colors (except blue) and postsynaptic densities (blue) were reconstructed from a set of 50 ATUM-SEM image stacks (40 nm/slice). **g**–**i** Synapse density from EM images in hippocampus (**g**), entorhinal cortex (**h**), and prefrontal cortex (**i**) quantified by the cumulative frequency graph. Insets display box plots of the actual synaptic density (number of synapses per 100 μm^2^). More than 5000 μm^2^ of images were analyzed in each group. *n* = 3 rats; *n* > 180 images/group. Statistics: Kolmogorov-Smirnov test (non-parametric test) was used to compare cumulative distributions.

Synapse degeneration is now regarded as an intermediate step and critical pathophysiological hallmark of AD.^8,58^ Substantial evidence indicates that in AD, there is a decrease in the number of synapses as well as impairments in synaptic functions, which occurs later than Aβ accumulation and correlates with disease progression.^58,59^ Thus, we investigated synaptic alterations in *App*^*NL-G-F*^ rats. Biochemical analysis by fractionation of synaptosomes from *App*^*NL-G-F*^ rat brains demonstrated a significant decrease in levels of synaptophysin and PSD-95, pre- and post- synaptic markers respectively, suggesting synaptic impairments in the *App*^*NL-G-F*^ rat brain (Fig. 4a–c). We also examined these synaptic markers in frozen sections of tissue, allowing for a *z*-axis resolution of approximately 30 μm. Significant changes were observed in synaptophysin and PSD-95 density, especially around the Aβ plaques in 9-month-old homozygous *App*^*NL-G-F*^ rats (Fig. 4d). Quantification of synaptophysin and PSD95 puncta in regions with no Aβ plaques demonstrated a similar magnitude of reduction in synaptic proteins (Supplementary information, Fig. S6a, b), as that seen in Western blot (Fig. 4b, c). Synaptic degeneration was also observed in the 12-month-old homozygous *App*^*NL-G-F*^ rats, as revealed by swelling and hollowing of postsynaptic density (PSD-95) in the homozygous brains (Supplementary information, Fig. S6c). To quantify the synaptic loss in *App*^*NL-G-F*^ rat brain, quantitative electron microscopy (EM) was also used to examine the density and structure of synapses in several brain regions most relevant to AD, including hippocampus (HPC), entorhinal cortex (EC) and prefrontal cortex (PFC). Serial sections were collected and imaged, and pre-synaptic elements were quantified using a previously reported method.^60^ There was a clear reduction in synaptic density in HPC (Fig. 4e, g), EC and PFC (Fig. 4h, i) in homozygous *App*^*NL-G-F*^ rat brains. To further assess the synaptic impairment in *App*^*NL-G-F*^ rats, we also quantified the area and perimeter for each synapse. The quantitative results indicate that the synaptic area and perimeter in these three brain regions were all significantly reduced in *App*^*NL-G-F*^ rats comparing with those in WT rats (Supplementary information, Fig. S6d–i). We also performed 3D reconstruction of EM images in the CA1 area. Postsynaptic densities were labeled in blue and the pre-synapses were labeled with other colors. The presynaptic atrophy and the shrinkage of postsynaptic elements were even better revealed in the 3D reconstruction images (Fig. 4f; Supplementary information, Fig. S6j, Video S1a, b). Finally, we also performed biochemical analysis by fractionation of synaptosomes from 3-month-old *App*^*NL-G-F*^ rat brains and found that the levels of synaptophysin and PSD-95 were not changed (Supplementary information, Fig. S6k–m), suggesting that the synaptic impairments of *App*^*NL-G-F*^ rats may start between the age of 3 to 6 months.

### Neuronal loss, brain atrophy, and increased apoptosis and necrosis in *App*^*NL-G-F*^ rats

**Fig. 5:**
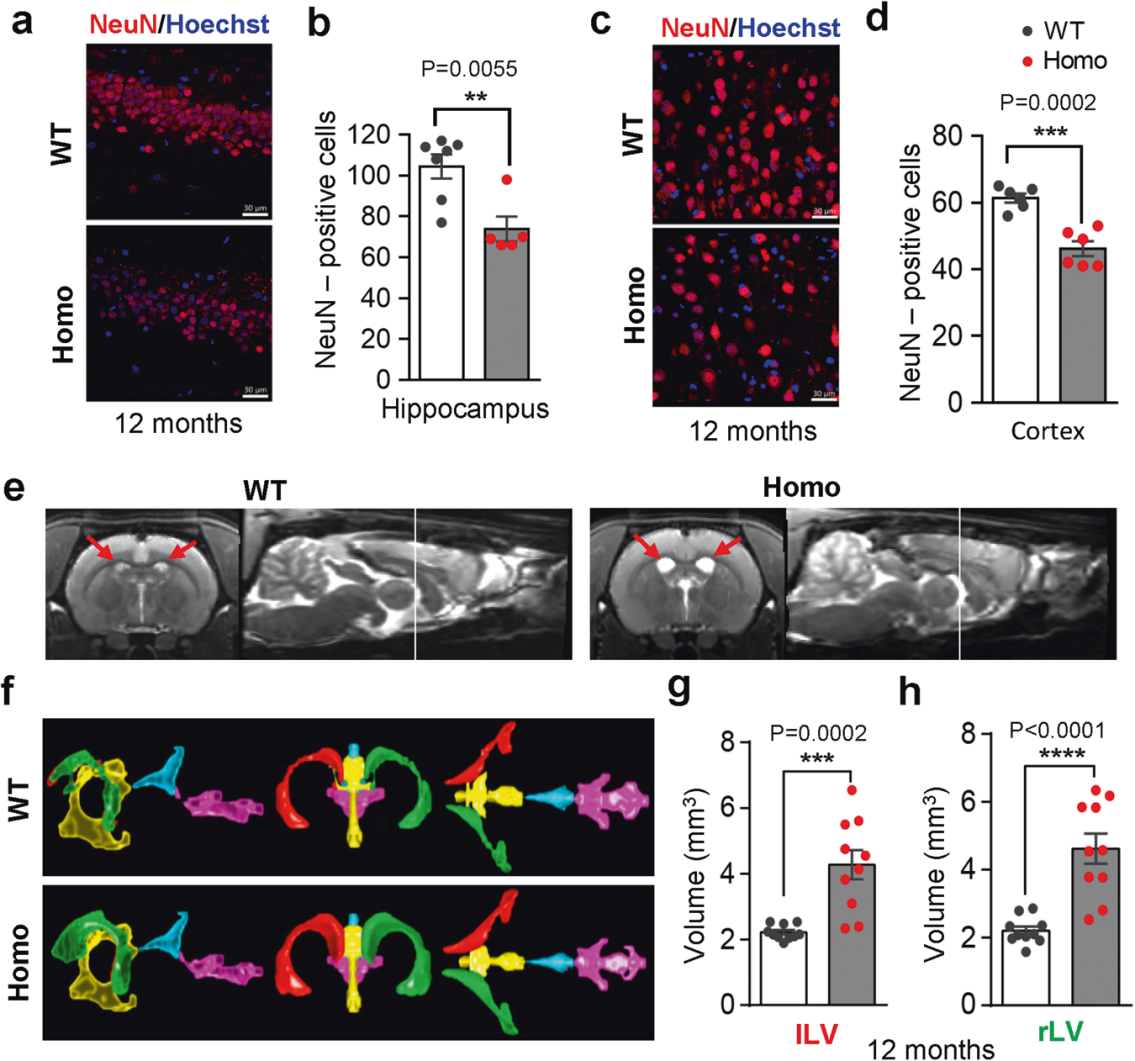
Neuronal loss and brain atrophy in *App*^*NL-G-F*^ rats. **a**, **d** Neuronal loss in the hippocampus and cortex of *App*^*NL-G-F*^ rats. Brain sections from 12-month-old WT or homozygous (Homo) rat were stained with an antibody for NeuN, a neuronal marker. Quantitations of NeuN-positive cells in the hippocampus and cortex of stained sections are shown in (**b**, **d**) respectively. *n* = 5 (Homo) or 7 (WT) rats for hippocampus. *n* = 6 rats for cortex. Scale, 30 μm. **e**–**h** Enlarged ventricles in *App*^*NL-G-F*^ rats. Representative MRI images showing the larger ventricles in a 12-month-old Homo rat compared with its WT counterpart (**e**). The bright regions indicated by arrows are the lateral ventricles. 3D reconstruction of ventricles in WT and Homo rats (**f**). Volumetric quantitation of the left and right lateral ventricles (**g**, **h**). *n* = 10 rats.

Brain atrophy caused by neuronal loss is another prominent pathological feature of AD.^61^ However, neuronal loss is not observed in either *App* transgenic or knock-in mice. Remarkably, the *App*^*NL-G-F*^ rats developed massive neuronal loss in the hippocampus, as shown by staining of hippocampal sections with an antibody for NeuN, a commonly used neuronal marker (Fig. 5a). Notably, the number of NeuN-positive neurons was decreased by approximately 30%, and 50% in the 12-, and 22-month-old homozygous *App*^*NL-G-F*^ rats respectively (Fig. 5b; Supplementary information, Fig. S7a, b). In addition, neuronal loss was examined in other brain regions. We also found significant loss of NeuN-positive neurons in cortex (Fig. 5c, d). In association with the neuronal loss and brain atrophy, AD patients exhibit enlarged ventricles.^62^ Using a 9.4 Tesla magnetic resonance imaging (MRI) scanner, we acquired T2-weighted anatomical images to quantify the volumetric difference in ventricle system. In coronal sections, an enlargement of the lateral ventricles was visible in 12-month-old homozygous *App*^*NL-G-F*^ rats compared with WT littermates (Fig. 5e; Supplementary information, Fig. S7c, d). Surface-rendered 3D reconstructions of the ventricle system were performed with the segmented MRI images of representative WT and homozygous *App*^*NL-G-F*^ rats. The enlargement of bilateral lateral ventricles was demonstrated by the three orthogonal views (Fig. 5f; Supplementary information, Video S2), as well as the quantification of the left and the right lateral ventricles, respectively (Fig. 5g, h). The size and weight of *App*^*NL-G-F*^ rat brains were also reduced (Supplementary information, Fig. S7e). Quantitative analysis revealed an overall brain weight loss of 9.11% and 16.09% in 12- and 22-month-old homozygous rats, respectively (Supplementary information, Fig. S7f). Therefore, the *App*^*NL-G-F*^ rats exhibit progressive neuronal loss and brain atrophy, similar to those seen in AD patients.

**Fig. 6:**
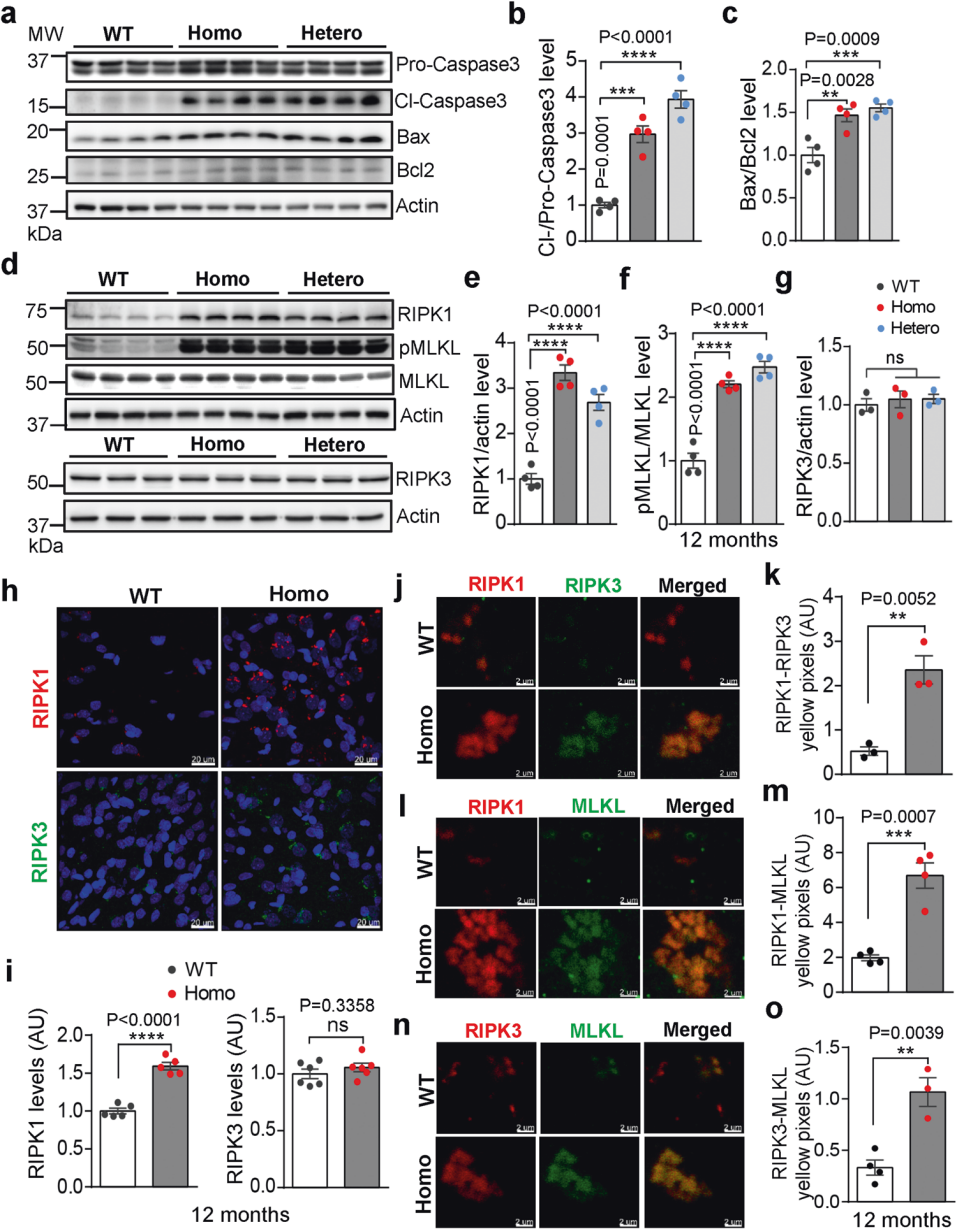
Increased apoptosis and necroptosis in *App*^*NL-G-F*^ rats. **a**–**c** Expression of apoptotic proteins in *App*^*NL-G-F*^ rats. **a** Hippocampal lysates from 12-month-old WT, Homo, and Hetero rats were immunoblotted using anti-cleaved caspase3, anti-procaspase3, anti-Bax and anti Bcl-2 antibodies. **b**, **c** Apoptosis was quantified by the ratios of cleaved caspase3 to procaspase3, as well as by the ratios of Bax to Bcl-2, shown in the right two panels. *n* = 4. **d**–**g** Expression of necrotic proteins in *App*^*NL-G-F*^ rats. **d** Hippocampal lysates from 12-month-old WT, Homo, and Hetero rats were immunoblotted using anti-RIPK1, anti-phosphorylated MLKL, anti-total MLKL (Upper panel), and anti-RIPK3 (Lower panel) antibodies. **e**–**g** The necroptosis levels were quantified by the ratio of RIPK1 to actin, the ratio of pMLKL to total MLKL, and the ratio of RIPK3 to actin. *n* = 4, Statistics: one-way ANOVA. **h**, **i** Representative fluorescent images of brain sections from WT and Homo rats stained with anti-RIPK1 and RIPK3 antibodies (**h**). Scale bars, 20 μm. Quantitative analyses of the immunoreactivity are shown below (**i**). *n* = 5. **j**–**o** Necrosomes in *App*^*NL-G-F*^ rats. Necrosome-like structures were immunostained by RIPK1, RIPK3 and MLKL antibodies. Representative fluorescent images and quantitative analyses of co-localization of necroptotic protein markers on the brain sections from 12-month-old WT and Homo rats are shown in the left (**j**, **l**, **n**) and right (**k**, **m**, **o**) panel respectively. Scale bars, 2 μm.

Cell death in AD brain has been attributed by at least two mechanisms: apoptosis and necrosis. Apoptosis can be measured by the ratios of BAX/Bcl-2 and cleaved caspase-3/pro-caspase3.^63^ We observed that apoptotic markers increased in *App*^*NL-G-F*^ rats at 6-month age (Supplementary information, Fig. S8a), and they became increasingly obvious at the age of 12-month (Fig. 6a). Quantitative analysis showed that the ratio of BAX/ Bcl-2 and that of cleaved caspase-3/pro-caspase3 were significantly higher in *App*^*NL-G-F*^ brains than in WT at the age of 6-month (Supplementary information, Fig. S8b, c) or 12-month (Fig. 6b, c), but not 3-month (Supplementary information, Fig. S8d–f). To examine whether apoptosis contributed to neuronal death, we performed NeuN/Cl-Caspase3 immunofluorescent double-labeling staining and found significant neuronal apoptosis in the brain sections from 12-month-old *App*^*NL-G-F*^ rats (Supplementary information, Fig. S8g). In addition, we also examined apoptosis in female homozygous rats. TUNEL-positive immunofluorescence was also observed in female *App*^*NL-G-F*^ rat brains at the age of 12-month (Supplementary information, Fig. S8h).

Unlike apoptosis, necrosis is an uncontrolled lysis of the cell. Necroptosis, a programmed form of necrosis was recently identified in postmortem AD brains.^64^ Three critical proteins, RIPK1, RIPK3 and its substrate MLKL, are involved in the initiation of necroptosis. Phosphorylated MLKL can trigger MLKL aggregates to form homodimers, which induce membrane damage that leads to cell death.^65^ To determine whether necroptosis was activated in *App*^*NL-G-F*^ rat brains, we measured the expression levels of RIPK1, RIPK3, phosphorylated MLKL (pMLKL) and MLKL in RIPA fractions. We found that the levels of RIPK1 and pMLKL were mildly increased in 6-month-old *App*^*NL-G-F*^ rats (Supplementary information, Fig. S9a–d) but markedly increased in 12-month-old *App*^*NL-G-F*^ brains (Fig. 6d–f), compared with the WT controls. Consistent with the previous report in AD patients, RIPK3 expression levels were not significantly different between *App*^*NL-G-F*^ and WT rats either at 6-month (Supplementary information, Fig. S9a, d) or 12-month of age (Fig. 6d, g), but not at 3-month of age (Supplementary information, Fig. S9e–g). We also measured the levels of RIPK1 and RIPK3 by staining sections and found that RIPK1 immunoreactivity was higher in homozygous *App*^*NL-G-F*^ rat brains than in 6- (Supplementary information, Fig. S9h, i) and 12-month-old WT rats (Fig. 6h, i). Similar to immunoblots results, there was no difference in RIPK3 expression level between the two groups (Fig. 6h, i; Supplementary information, Fig. S9h, j). Further, we found that most of the RIPK1 immunoreactivity was detected in NeuN-positive neurons in 12-month-old *App*^*NL-G-F*^ brains (Supplementary information, Fig. S9k, l).

During the activation of necroptosis, RIPK1 binds to and activates RIPK3 to form necrosomes, which then recruit and activate MLKL.^65^ To determine whether these events occur in *App*^*NL-G-F*^ rat brains, we measured necrosome-like structures by immunostaining of RIPK1, RIPK3 and MLKL. The representative images and quantitative results showed significantly higher degree of RIPK1 and RIPK3 colocalization (Fig. 6j, k), RIPK1 and MLKL (Fig. 6l, m) colocalization and stronger MLKL-RIPK3 (Fig. 6n, o) interaction in brain sections from *App*^*NL-G-F*^ brains compared with those in WT brains at the age of 12-month. These observations together suggest that both apoptosis and necroptosis may contribute to neuronal loss in *App*^*NL-G-F*^ rats.

### Comparison of *App*^*NL-G-F*^ rats with *App*^*NL-G-F*^ mice

To compare the pathologies of the *App* knock-in rats with the previously generated *App* knock-in mice^19^ and to explore the potential underlying mechanisms, we performed an age-matched comparison study with the *App*^*NL-G-F*^ mice (12-month-old, homozygous), using identical reagents and experimental protocols. First, we examined the Aβ plaques and tau phosphorylation in *App*^*NL-G-F*^ mice brains at different ages. Using a specific antibody for Aβ (82E1), Aβ depositions in homozygous *App*^*NL-G-F*^ mouse brains were detected and quantified in hippocampal, cortical and sub-cortical regions. The fluorescent images and quantification curves were similar to those of *App*^*NL-G-F*^ rats (Fig. 1f, g; Supplementary information, Fig. S10a, b). Tau phosphorylation was also examined with the same antibodies used in rat experiments. Both AT180/Tau5 and AT8/Tau5 levels were very low in mice. However, the statistical analysis showed a small but significant increase in AT8/Tau but not AT180/Tau5 in *App*^*NL-G-F*^ mice compared with WT mice. (Supplementary information, Fig. S10c–e). Similar results were also reported in previous publication, in which showed that a mild increase of Tau phosphorylation in AT8/Tau5, mainly depends on a slight reduction of Tau5.^66^ Next, neuroinflammation was assessed by the analysis of the astrocytic marker GFAP and the microglia marker Iba1 (Supplementary information, Fig. S11a–c), both of which were significantly increased in *App*^*NL-G-F*^ mice, reflecting the robust neuroinflammation in the brains of these mice. Furthermore, synaptic alterations were found as reflected by decreased PSD95 levels in *App*^*NL-G-F*^ mice, whereas synaptophysin levels remained unchanged (Supplementary information, Fig. S11d–f). In contrast to the *App*^*NL-G-F*^ rats, analysis of apoptotic markers Bax/Bcl2 and cleaved caspase 3 did not reveal any sign of apoptosis (Supplementary information, Fig. S11g–i). Similarly, markers for necroptosis including RIPK1, RIPK3 and MLKL/pMLKL were not altered in the *App*^*NL-G-F*^ mice indicating absence of necroptosis (Supplementary information, Fig. S10j–m).

### Cognitive deficits in *App*^*NL-G-F*^ rats

**Fig. 7:**
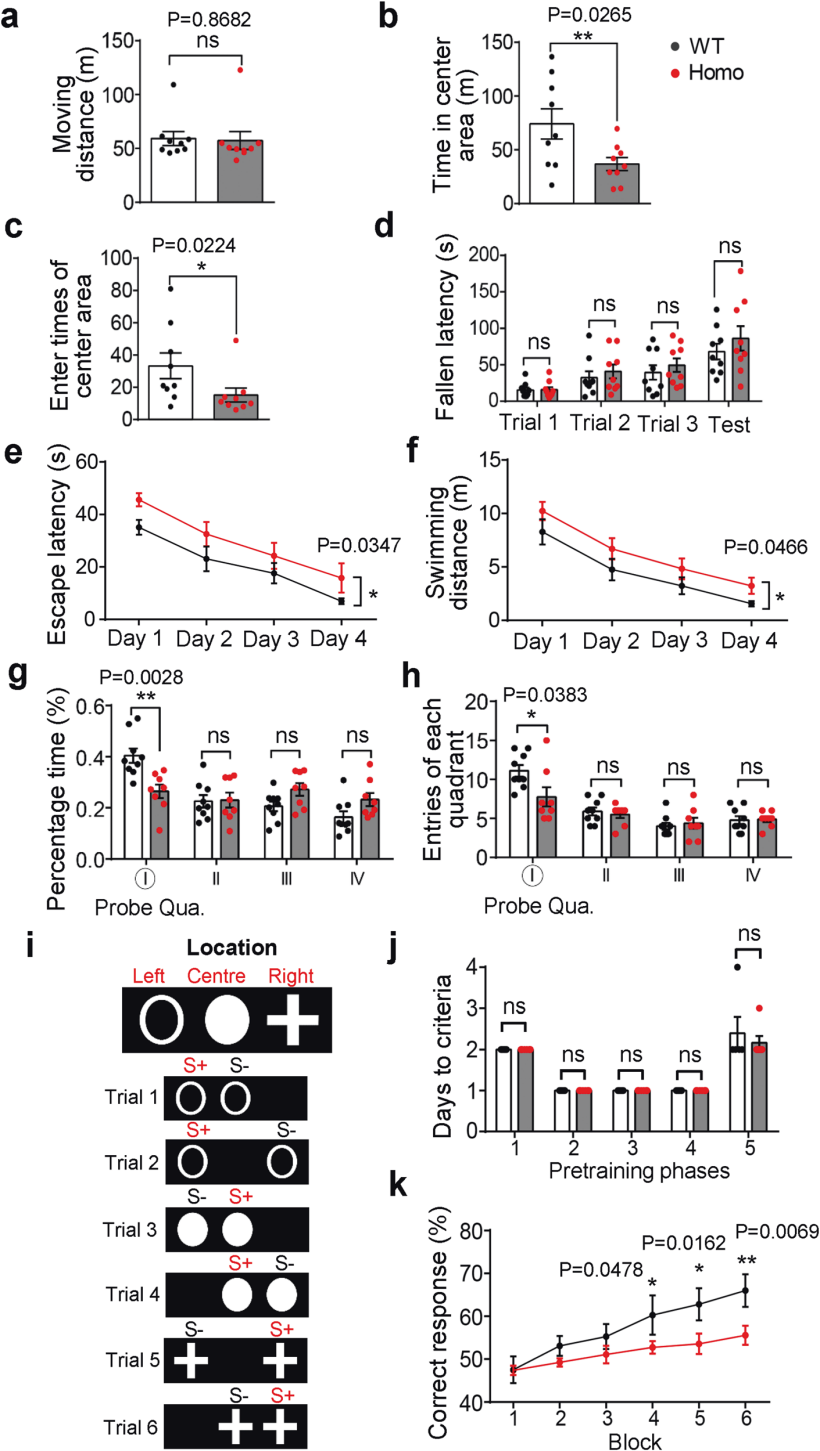
Cognitive deficits in *App*^*NL-G-F*^ rats. WT and *App*^*NL-G-F*^ male rats of 5 months old were used for behavioral experiments. **a**–**c** Horizontal locomotor activity measured using the open field test. Locomotion within 10 min after habituation was measured: (**a**) total distance traveled; (**b**) time spent in the central area; (**c**) the number of entries to the central area. *n* = 9/group. Note that the Homo rats appeared to avoid the center area. **d**, Motor coordination using rotarod test. The time on the rotarod before the rat fell was recorded. *n* = 9/group. **e**–**h** Spatial learning and memory. WT and Homo rats were subjected to learning paradigm of water maze for 4 days (**e**, **f**), followed by a probe trial at day 5 (**g**, **h**). Escape latency (**e**) and distance (**f**) of learning trials. Probe tests were conducted to evaluate spatial memory. The times spent in each quadrant (**g**) and entries of each quadrant (**h**) of probe trials are presented. *n* = 8–9/group. Statistics: two-way RM ANOVA for spatial learning, and *t*-test for probe test. **i**–**k** PAL test. WT and Homo rats (*n* = 9) were subjected to PAL test for 30 days with 100 trials per day after pretraining and training. **i** Touchscreen objects and locations. The animals were trained to associate three different stimuli (hollow circle, solid circle and cross) with three specific locations (left, center and right) (upper). There were all together 6 trial types in the PAL task (lower). **j** Time (days) to criterion in pre-training stage of 5 different phases. No difference was found between WT and Homo rats. **k** Mean percentage of correct response in testing stage. Each block represents 5 consecutive testing days. Statistics: two-way RM ANOVA with LSD post hoc, significant genotype and time interaction effect, *P < 0.05; **P < 0.01, compared with WT group. Note that the WT rats performed significantly better than the *App*^*NL-G-F*^ rats.

To ensure proper assessment of the cognitive impairment, we first examined sensorimotor functions using open field test and rotarod test, at time points shown (Supplementary information, Fig. S12a). In open field test, the homozygous *App*^*NL-G-F*^ rats had fewer number of entries to the central area and spent less amount of time there than their WT littermates (Fig. 7b, c), whereas the total distances traveled within the open field were the same between the two genotypes (Fig. 7a). This result suggests that the *App*^*NL-G-F*^ rats have a mild anxiety, while their locomotor activity is normal. In the rotarod test, there was no difference between *App*^*NL-G-F*^ and WT rats in the falling latencies during the 3 trial sessions and the test session (Fig. 7d), suggesting that the motor coordination of the *App*^*NL-G-F*^ rats is intact.

Cognitive dysfunction in AD, due largely to synapse loss and neuronal death, is manifested as impairments in spatial learning memory. To measure spatial learning and memory, we performed Morris water maze (MWM) test. In the learning sessions, the *App*^*NL-G-F*^ rats displayed longer latency and swimming distance to reach the hidden platform in quadrant I (QI) than WT controls as early as 5-month of age (Fig. 7e, f). Thus, the *App*^*NL-G-F*^ rats are impaired in their spatial learning capacity. The swimming speed was not different between the two genotypes (Supplementary information, Fig. S12b), again suggesting normal sensorimotor functions. In the probe test, the *App*^*NL-G-F*^ rats spent less amount of time in the targeted quadrant QI (Fig. 7g). The mutant rats also exhibited fewer number of entries to QI (Fig. 7h). These results together point to a clear deficit in spatial memory in the *App*^*NL-G-F*^ rats at 5-month of age, which is consistent with the data that synaptic deficits happened between 3- to 6-month old (Fig. 4; Supplementary information, Fig. S6).

A more typical phenotype in AD patients is the deficits in episodic memory, a recollection of previous experiences together with their context in terms of time, place, associated emotions, etc.^67^ This form of memory could be measured in rodents using paired associates learning (PAL) task.^68^ The rats were subject to pretraining, training and testing stages as outlined in Supplementary information, Fig. S12c. During the pretraining stage, rats from the *App*^*NL-G-F*^ and WT groups showed no differences in the time required to reach criterion in each phase (Fig. 7j), indicating that *App*^*NL-G-F*^ rats did not present any major visual impairment and had normal learning ability to associate image with rewards in the operant conditioning chambers (Supplementary information, Fig. S12d). In the training stage, rats were trained to learn association rules of the three different stimuli (hollow circle, solid circle and cross) in three corresponding locations (left, center and right)) on touchscreens, with a total of six possible trial types (Fig. 7i). After the training sessions, the animals were tested for their ability to do the paring. A trial was scored as correct if a nose-poke occurred at a right stimulus in the right location on the touchscreen. In WT rats, the percentage of correct response, defined as “correct responses/(correct responses + incorrect responses) × 100”, continued to increase over time (Fig. 7k). In contrast, the *App*^*NL-G-F*^ rats presented a continuously lower percentage of correct response than WT rats during a course of 30-day PAL test, indicating that object-location associates learning ability was significantly impaired in *App*^*NL-G-F*^ rats (Fig. 7k).

Taken together, these data indicate that both the spatial learning and memory and the episodic-like memory are impaired in the *App*^*NL-G-F*^ rat model.

## Discussion

Over the last two decades, more than 200 animal models for Alzheimer’s disease have been developed, but few has been viewed as adequately recapitulating the full or key pathological features of AD. For Aβ-based mouse and rat models, in addition to non-physiological phenotypes possibly derived from overexpression and mis-expression of APP and its fragments, the key shortcomings in most of these models are the lack of tau pathology and cell death. These shortcomings have limited their applications in mechanistic studies and drug testing. Here by introducing a single chimeric *App* gene through a knock-in strategy, we were able to construct multiple AD pathologies in a rat model. First, the expression of chimeric *App* gene is driven by the rat endogenous promoter, averting the problem of overexpression of full-length APP and APP proteolytic fragments. Second, this model displays comprehensive Aβ pathology and neuroinflammation including astrocytosis and gliosis. Third, tau pathology was observed in this rat APP model, while it is rarely seen in other *App* animal models. Most importantly, this rat model exhibits neuronal death mediated by both apoptosis and necroptosis, leading to a severe atrophy of cerebral cortex including enlargement of ventricles. Further, these pathological changes (Aβ, gliosis, tau, synaptic degeneration, memory deficits) appear to occur in a time sequence similar to those seen in human AD patients. Thus, this new rat model appears to recapitulate most of the pathologies observed in human AD brain. The *App*^*NL-G-F*^ knock-in rats may facilitate investigations to delineate the interaction between different disease mechanisms, and allow head-to-head comparison of molecules targeting different pathways in a single preclinical model to prioritize for clinical testing.

Neuronal loss is a hallmark of AD contributing to cognitive and functional decline. As a consequence, brain atrophy can be detected in the brains of AD patients by imaging or postmortem examination.^61^ Extensive studies have revealed a substantial neuronal apoptosis throughout human AD brain, particularly in the cerebral cortex.^69^ Recent studies have shown that in addition to apoptosis, necroptosis is widespread in the AD brain as well.^64^ However, few mouse models with APP or APP/PSEN mutations exhibit neuronal loss. In the mouse transgenic APP models, such as APP23 (Swedish), J20 (Swedish/Indiana), APPswe/PSEN1dE9 (Swedish mutation and human PSEN1 lacking exon 9), APP751SL/PS1 KI (transgenic of Swedish/London mutations plus knock-in of two PS1 mutations), neuronal loss is not detectable until very old ages, primarily limited to the CA1/CA2 region of the hippocampus or adjacent to Aβ plaques.^70–72^ The 5× FAD model shows cell death in a number of brain regions at 9-month of age.^16,73–75^ In the few transgenic rat AD models, neuronal loss could also be observed at 18-month of age.^27,36,76^ However, overexpression of the transgene makes it difficult to interpret the results from all these transgenic animals. Some transgenic models expressing human wild type or mutant *tau* also exhibit cell death.^77,78^ However, the cell death could be detected in areas not relevant to AD, such as spinal cord.^78^

In a comprehensive review, Sasaguri et al. have termed these the “first-generation mouse models”, and summarized many limitations caused by Aβ overexpression.^18^ To overcome the limitations of transgenic models, the “second generation” knock-in mice have been generated. Mice with a single FAD gene mutation knocked into their genome show either no phenotype or mild phenotypes till 2 years after the Aβ deposits are clearly seen.^79–81^ The *App*^*NL-F*^ (harboring the Swedish and the Beyreuther mutations) and *App*^*NL-G-F*^ (harboring the Swedish, the arctic and the Beyreuther mutations) knock-in mice do exhibit Aβ plaques from 9- and from 3-month of age, respectively.^19^ However, no neuronal death has been observed in these mice. In comparison, the *App*^*NL-G-F*^ rat model generated in this study showed an obvious brain atrophy, with larger ventricular cavity and brain weight loss beginning at the age of 6-month of the animals. Biochemical and immunostaining analyses indicate that both apoptosis and necroptosis contribute to the neuronal loss in the AD rats. These notable markers for cell death could be detected as early as 6-month of age. As far as we know, this may be the only *App* knock-in animal model with significant neuronal apoptosis and necroptosis as well as brain atrophy.

Formation of neurofibrillary tangles due to pathological aggregation of tau protein is another hallmark of AD. Unfortunately, none of the mouse models with APP or APP/PSEN1 mutations reported so far exhibits tau pathology. Transgenic mice carrying human *tau* mutations derived from frontotemporal dementia (FTD) such as P301L and A152T have been generated.^82–84^ These mice exhibit hyperphosphorylation of tau in many residues, aggregated tau protein, and even neurofibrillary tangles.^82,83^ However, no plaques or any of the Aβ pathology have been found in these transgenic tau animals.^82–84^ It should also be pointed out that *tau* mutations have only been found in frontotemporal lobe degeneration (FTD) with Parkinsonism linked to chromosome 17 (FTDP-17) but not in Alzheimer’s patients.^85^ Thus, the link between Aβ pathology and tau pathology seen in human is missing in these mouse models: an absence of tauopathy in Aβ-based AD models, and absence of Aβ pathology in mutant tau-based AD models. This is in marked contrast with the situation in human. Both Aβ plaques and tau-containing neurofibrillary tangles are prominently seen in the brains of sporadic AD patients. In familial Alzheimer Disease (FAD) patients with mutations in the *App* or *Psen1* gene, tau pathology has also been observed.^86^ Interestingly, no Aβ pathology has been found in patients with *tau* mutations such as FTD. While it has been postulated that tau pathology is necessary for Aβ-induced neurotoxicity,^87^ whether tau protein lesions are secondary to Aβ deposition or an independent disease phenotype remains controversial.^5,88^

An important finding of the present study is the presence of some tau phenotypes in the *App*^*NL-G-F*^ rat model. In 12-month-old brains, tau protein was found to be significantly hyper-phosphorylated in Thr231 and Ser202, two hyper-phosphorylation sites commonly seen in AD patients. An early-phase tau aggregation was also determined by a newly generated antibody APN-mab005, which specifically recognize aggregated but not monomeric tau,^52^ in 12-month-old brains. In aged *App*^*NL-G-F*^ rat model, MC1 antibody also detected conformationally altered tau, a marker for pathological aggregation of tau protein. A neurofibrillary tangle-like pathology was also labeled by APN-1607, a tracer for aggregated tau.^54,55^ Interestingly, the tau phenotypes seem to occur much later than that of Aβ in our rat model. We detected Aβ deposits as early as 3-month of age, but the earliest time we could find hyperphosphorylation of tau was in 6-month-old brain, and thioflavin-S positive staining was observed at the age of 22-month, albeit they did not appear typical NFT-like structures. Consistent with our finding, some tau pathologies were also found in some of the amyloid transgenic rat models, as well as rats transfected with exogenous App and Psen1 mutations by AAVs.^26,89^ These results seem to support the notion that accumulation of Aβ plaques could lead to hyperphosphorylation and even aggregation of tau in rats but not mice, suggesting that the genomic environment of rats is more resemblance to that of human, compared with mice. Further experiments are necessary to validate this point and delineate its underlying mechanisms.

To ensure the tau phenotypes are truly unique in rats, we performed side-by-side comparison between our *App*^*NL-G-F*^ rats and the *App*^*NL-G-F*^ mice (carrying the same mutations and humanized Aβ sequence), using identical age, reagents and experimental protocols. Interestingly, tau hyperphosphorylation was difficult to detect in the *App*^*NL-G-F*^ mice (Supplementary information, Fig. 10c–e). Given that the APP mutations as well as the design of knock-in genomic constructs are identical between the two species, why would *App*^*NL-G-F*^ rats show tau phenotypes whereas the *App*^*NL-G-F*^ mice do not? One idea is that the isoforms of tau in different species are different. Tau isoforms containing three repeat regions (3R) are mainly expressed in human, mouse and rat at the early stage of development.^90^ In adult rodents, the isoforms containing four repeat regions (4R) gradually increase and the expression of 3R isoforms is downregulated. The proportion of 3R and 4R isoforms in human is about 1:1. Importantly, there is no 3R isoform in adult mice. In contrast, there are still a substantial amount of 3R isoform in adult rats, and the ratio of 3R to 4R is about 1:9.^24,90^ In the brains of AD patients, 3R and 4R isoforms seem to be equally present in less severe cases. However, more 3R isoforms were detected in AD brains of severe stage.^91^ It is therefore tempting to speculate that the tau isoforms in rats are closer to those in human than mice, and thus more likely to produce tau pathology upon amyloid loading. Consistent with this, transgenic rats over-expressing APP also display significant tau pathologies.^26^ It should be pointed out that the current study has only assessed *App*^*NL-G-F*^ mice in one genetic background (C57BL/6). It is unclear whether *App*^*NL-G-F*^ mice with different genetic backgrounds would display tau phenotypes.

Using PET imaging for microglial activation ([^11^C] PBR28), Aβ ([^18^F] AZD4694) and tau ([^18^F] MK-6240), Pascoal et. al studied the relationships of the three key pathological features in AD. They showed that [^11^C] PBR28 signal/CSF TREM2 (a microglia marker) and [^18^F]MK-6240 correlated hierarchically following Braak-like stages, suggesting that microglial activation sets the stage for the stereotyped spread of tau in Braak stages in AD patients. Further, they found that Aβ potentiates the effects of microglial activation on tau spreading.^92^ More importantly, co-occurrence of Aβ, tau and microglia abnormalities seems necessary for a full-bloom dementia phenotypes.^92^ These results, while correlative, are consistent with our finding that Aβ deposites occurred first, and microglial activation and tau pathology co-increase from 6-month to 12-month in the *App*^*NL-G-F*^ rat model.

The cell death phenotypes were observed in the rat model, and the mouse model with the same mutations and same genomic design (*App*^*NL-G-F*^ knock-in) do not show any feature of cell death, neuronal loss or brain atrophy.^19^ Side-by-side comparison revealed a pronounced neuronal apoptosis and necroptosis as well as brain atrophy in 12-months-old rat *App*^*NL-G-F*^ model. In contrast, neither apoptosis nor necroptosis was detected in the mouse *App*^*NL-G-F*^ model, using identical age, reagents and experimental protocols (Supplementary information, Fig. S10g–m). Interestingly, the rat and mouse models are very similar in the progressive accumulation of Aβ plaques, as well as the degree of gliosis including both astrocytosis and microgliosis. These results together may lead to a hypothesis that Aβ accumulation and gliosis are not sufficient to cause neuronal death. Given that there is tauopathy in the rat but not mouse models, one may speculate that alteration in tau (hyperphosphorylation, aggregates, etc) may be more relevant to neuronal death in AD. Furthermore, one school of thought suggests that Aβ-induced neuronal death is mediated by hyperphosphorylation and aggregation of tau.^87^ However, tau pathology may not be the only factor required for neuronal death. A recent study reported that introduction of humanized tau gene into the genome of the *App*^*NL-G-F*^ mice resulted in tau hyperphosphorylation, but these mice still exhibit no neuronal loss.^66,93^ Further analyses of the mouse and rat *App*^*NL-G-F*^ models may help delineate the relationships between Aβ, tau and neuronal death.

The distinctive features seen in our *App*^*NL-G-F*^ rats but not the *App*^*NL-G-F*^ mice also support the notion that rats may have a unique place in modeling human diseases, especially neurodegenerative disorders. In addition to AD animal modeling, severe dopamine loss and motor behavior deficits induced by overexpression of α-synuclein only could be reproduced in PD rat model.^37^ Deletion of Pink1 in rats but not in mice exhibit critical pathologies of α-synuclein aggregates and dopamine neuronal death.^38,94^ More comprehensive phenotypes in transgenic rats have been also shown in modeling atherosclerosis.^95^ Moreover, rats are more similar to human in physiology and behaviors than mice. The comparisons in aging pathologies including metabolic, cardiovascular and nervous systems between rats and mice demonstrate that rats are more appropriate to model aging-related diseases.^96^ Finally, the larger brain volume of rat makes it easier for brain surgery, in vivo electrophysiological recording, optogenetics, imaging studies, and so on. Rats are more suitable for complex behavioral studies, especially those relevant to AD. With their bigger body size rats have the volumes of blood and cerebrospinal fluid (CSF) that are 5–10 times larger than those in mice. It is possible to perform more collections or larger volume of blood in rat than in mouse at different time points in the same animals.^97^

An interesting phenomenon was that the female *App*^*NL-G-F*^ rats showed a more aggressive Aβ pathological progress than the males (Supplementary information, Fig. S2d, e). This is remarkably similar to what has been observed in human: there are more women with AD than men (about two-thirds of AD patients are women).^98^ In addition to the fact that women live longer than men (aging is the biggest risk factor for AD), several factors may contribute to a higher risk of AD in women: First, age-related loss of sex hormones may increase the risk for AD.^99^ Second, some risk genes, such as ApoE4, may affect women more than men.^100^ A sex-stratified GWAS with 5976 participants showed that genetic factors act in a sex-specific manner to drive AD pathologies.^101^ Third, tau pathologies may spread more in women than in men, particularly in MCI patients.^102^ Our *App*^*NL-G-F*^ rat model may be highly useful in studying sex differences in AD and their underlying mechanisms.

As a new model, the *App*^*NL-G-F*^ rats have been characterized in several key aspects of pathologies. However, there are still lots of unknowns. It has not been determined whether these rats also exhibit other pathological changes such as metabolic abnormalities, vascular changes and BBB injury. In addition, although the model exhibits significant Aβ and tauopathy, whether the spatial distribution and progression of these pathologies are comparable to those of AD patients still needs further identification. Most importantly, although this model using knock-in strategy is very close to the physiological status, it is almost impossible for AD patients to harbor several pathogenic *App* mutations. Future studies should be designed to address these questions. In particular, it would be of great interest to compare the transcriptomic, proteomic, and metabonomic profiles of the rat model with those of AD patients or popular mouse AD models.

In summary, the *App*^*NL-G-F*^ rat model appears to express some distinctive features compared with the existing rodent AD models, and may therefore be useful in many aspects of future research. Table 1 summarizes the relative timing for occurrence of various pathological features in the *App*^*NL-G-F*^ rat model, illustrating age-dependent progression patterns similar to that seen in human. This model offers a unique opportunity to address a number of important issues that are difficult to address otherwise. First, this new model with appropriate timing of pathologies opens up real opportunities to study how aging leads or affects neurodegeneration. Second, the *App*^*NL-G-F*^ rats could be used to investigate whether and how Aβ accumulation alone could lead to tau pathology in AD. It should allow us to address an important and yet unanswered question in the AD field: whether tau protein aggregation is secondary to Aβ deposition, or an independent factor contributing directly to neurodegeneration. Third, apoptosis and necroptosis are both present in the *App*^*NL-G-F*^ rats. Therefore, with this model, we may also study the difference and relationship between these two types of cell death, as well as their underlying mechanisms. Finally, compared with mouse models, the *App*^*NL-G-F*^ rat model offers some unique advantages, as highlighted above. It is therefore more conducive to carry out pharmaceutical studies, biomarker finding, and various omics, using the *App*^*NL-G-F*^ rat model.

**Table 1:** Timing for occurrence of various pathological features in the *App*^*NL-G-F*^ rat model.

Pathologies/Age (month)	1	2	3	6	12	22
Aβ oligomers	+	++				
Aβ plaques	+/−	+	++	+++	++++	+++++
Tau hyperphosphorylation			−	+	++	
Tau aggregation (APN-mab005)					+	++
Tau conformational change (MC1)						+
Tau aggregates (APN-1607)						+
Tau aggregates (Thioflavin-S)						+
Gliosis			−	+	++	
Synaptic loss			−	+	++	
Apoptosis			−	+	++	
Necroptosis			−	+	++	
Neuronal loss					+	++
Brain atrophy (MRI)					+	
Cognition impairment (MWM)				5–6 Months		
Cognition impairment (PAL)					7–8 Months	

*+* Positive, *−* Negative, *+/−* Very weak signals, *Blank:* not examined

## Materials and methods

### App^*NL-G-F*^ rats

Wild-type SD (Sprague Dawley) rats and *App*^*NL-G-F*^ rats were bred in the animal facility of Tsinghua University. All rat experiments were carried out according to the recommendations of AAALAC (Association for Assessment and Accreditation of Laboratory Animal Care International). The IACUC (Institutional Animal Care and Use Committee) of Tsinghua University approved animal protocol (15-LB5) used in this study. Rats were maintained on a standard 12 h light/12 h dark cycle, and were housed in groups of one to two. Food and water were provided ad libitum unless otherwise noted.

### App^*NL-G-F*^ mice

*App*^*NL-G-F*^ mice were previously generated using the same *App* genomic mutation design as the *App*^*NL-G-F*^ rats, and have been described in detail previously.^19^ Breeding, aging and handling of the mice at Karolinska Institutet were under ethical permission Dnr 15758-2019 (Stockholm’s animal research ethical board, Sweden). Mice were maintained on a standard 12 h light/12 h dark cycle and were housed in groups up to five. Food and water were provided ad libitum.

### Generation of App knock-in rat

Rat App genomic DNA was isolated, which included exon 16, intron 16 and exon 17. Then, the Aβ sequence in the rat APP was humanized by introducing mutations leading to the substitutions G676R, F681Y, and R684H. Furthermore, Swedish double mutations (K670N substitution and M671L substitution) were introduced into exon 16, and Beyreuther/Iberian (I716F substitution) and Arctic (E693G substitution) mutations were introduced into exon 17. Fig. 1a provides a scheme indicating the mutations introduced to obtain the modified rat APP. The mutated nucleic acid sequences were confirmed by DNA sequencing, as well as by enzyme digestion. The donor nucleic acid molecule comprises a 5′ homologous arm of 889 bp upstream of exon 16, and a 3′ homologous arm of 902 bp downstream of exon 17. The target gene sequence (i.e., target App gene sequence) may be different in different rat strains. Thus, to make sure of the efficiency of Cas9/sgRNA and to ensure consistent DNA sequences between sgRNA target and the sequences obtained from rat tail targets, PCR and DNA sequencing were performed with genomic DNA obtained from rat tails. The sequences obtained from the PCR products obtained from the rat tail DNA were the same as those recorded in the NCBI and Ensembl databases. SgRNAs were designed to be around the insertion sites (which were in the intron regions), and therefore insertion of heterologous fragments containing enzymatic restriction sites is easy to identify. Then, sgRNAs were ligated into a vector with T7 promoter, respectively, to obtain in vitro transcribed sgRNAs to be used in microinjection. Similarly, Cas9 mRNA was transcribed with T7 RNA polymerase in vitro. T7-Cas9 PCR products were gel purified and used as the template for in vitro transcription with the MEGAshortscript T7 kit (Life Technologies) according to the kit protocol. Cas9 mRNA was purified using the MEGAclear kit and eluted with RNase-free water.

The in vitro transcribed Cas9 mRNA, sgRNA2 and sgRNA6, as well as the APP targeting vector comprising the donor nucleic acid molecule were injected into the rat zygotes. The injected zygotes were then transplanted into the pseudopregnant recipient. To verify the correct recombination in F1 rats, Southern blotting was performed and at the same time 5′ Probe and A-Probe were used for further confirmation. A scheme of the gene editing strategy and the results of Southern blot analysis are shown in Supplementary information, Fig. S1a. The genotypes of the F1 rats were also determined using PCR, and DNA sequencing shown in Supplementary information, Fig. S1b. The cocktail of PCR primers is as follows: 5′-CTCACTCTCTGAGCAGAAGCCATGC-3′, 5′-TATGCACAGAGCCTGCTTCCGTGC-3′, 5′-CGAGTCCCTGCAAACTTCTATCTCGG-3′ and 5′-GGTCTTGCCTGTGTACTGAGCATGTG-3′.

### Immunofluorescence staining

Rats were perfused first with PBS followed by 4% paraformaldehyde under deep anaesthesia, and the brains were post-fixed overnight in 4% paraformaldehyde. Brains were sectioned at 30 μm using a vibratome (Leica). Sections were permeabilized and blocked in PBS containing 0.2% Triton X-100 and 10% normal goat serum at room temperature for 2 h. Sections were incubated overnight at 4 °C with the following primary antibodies: anti-Aβ antibody (1:1000, Cell Signaling Technology, 2454), 6E10 (1:1000, Covance, 39320) anti-Aβ oligomer antibody (OMAB) (1:1000, Agrisera, AS10932), anti-RIPK1 (1:200, BD Biosciences, 610459), anti-RIPK3 (1:500, Stata Crus, 374639), anti-MLKL (1:200, EnoGene, E11-11361C), anti-pMLKL (1:200, Abcam, ab196436), PSD95 (1:500, NeuroMab, 73-028), Synaptophysin1 (1:400, SYSY, 101002), MC1 (1:20; a gift from P. Davies, Albert Einstein College of Medicine, New York, New York, USA), anti-GFAP (1:1000, Millipore, MAB3402), anti-Iba1 (1:1000, Wako, 019-19741). 1-fluoro-2, 5-bis (3-carboxy-4-hydroxystyryl) benzene (FSB) was used for detection of amyloidosis. Next day, the sections were washed 3 times in PBS, and incubated with the appropriate secondary fluorescent antibodies for 1 h at 25 °C (AlexaFluor goat anti-mouse IgG (1:500, Invitrogen) or Alexa Fluor goat anti-rabbit IgG (1:500, Invitrogen). Cell nuclei were visualized with Hoechst 33342 (1:5000, Sigma-Aldrich; 94403) or DAPI (1:5000, Sigma-Aldrich; D9542).

For APN-1607 (a PET tracer specific for aggregated tau) staining, the brain sections were blocked in PBS containing 1% BSA and 10% normal goat serum for 2 h at room temperature with gentle shaking. After rinsing with 50 mM Tris-HCl buffer (pH7.4) for 1 min, the sections were transferred into the APN-1607-containing wells and incubated for 1 h at room temperature. Then the sections were washed 3 times in PBS with gentle shaking, and transferred onto the micro slide with mounting medium, followed by sealing with nail polish and air-drying. The sections were imaged on a Nikon A1 confocal microscope. Images were quantified using Image J and Imaris8 software and analyzed with Graphpad version 8.

### Thioflavin-S staining

For the assessment of NFT-like structure in brain, a series of paraffin sections was mounted and stained with Thioflavin S (T1892, Sigma). Coronal brain sections were subjected to Thioflavin S staining. In brief, brain sections were incubated in filtered 1% aqueous Thioflavin S for 8 min at room temperature followed by washing in 80% ethanol and 90%ethanol.

### TUNEL staining

TUNEL assay was performed using the In Situ Cell Death Detection Kit Fluorescence (Roche Applied Science, Mannheim, Germany). Postfixed frozen tissue sections were rinsed with PBS for 5 min and treated with 1% Triton X-100 in PBS for 2 min. Slides were rinsed in PBS for 5 min and then incubated for 60 min at 37 C with 50 μl of TUNEL reaction mixture. After being washed with PBS for 10 min, the slides with coverslips were analyzed with a laser scanning confocal microscope.

### Western blotting

Brain Tissue was homogenized in RIPA (50 mM Tris HCl pH 8.0, 150 mM NaCl, 1% NP-40, 0.5% sodium deoxycholate, CA630, 1% EDTA, 0.1% SDS) buffer with protease inhibitors (Roche Diagnostics). After a brief sonication (2× pulses for 10 sec at 30% intensity), tissue debris was isolated and discarded by centrifugation at 14,000 rpm. for 15 min. Lysates were quantitated using BCA protein assay kit (Thermo Fisher), and equal amounts of protein were loaded on SDS–PAGE gels. Protein was transferred from acrylamide gels to PVDF membranes (Immobilon-P, Millipore) at 90 V for 100 min. Membranes were blocked using bovine serum albumin (BSA, 5% w/v) diluted in TBS-T for 1 h. After blocking, membranes were incubated overnight at 4 °C in BSA (2.5% w/v) in TBS-T with the appropriate primary antibodies: anti-Human Aβ1–12 (6E10, 1:1000, Covance, 39320), anti-APP-CTF (1:1,000, Sigma, A8717), anti-APP N-terminus (1:1000, Millipore, 22C11), anti-Caspase3 (1:1000, Cell Signaling Technology, 9665 s), anti-Bax (1:1000, Abcam, ab32503), anti-Bcl-2 (1:500, Stata Crus, 7382), anti-RIPK1 (1:1,000, BD Biosciences, 610459), anti-RIPK3 (1:500, Stata Crus, 374639), anti-MLKL (1:1000, EnoGene, E11-11361C), anti-pMLKL (1:1000, Abcam, ab196436), anti-Human PHF-tau(AT180) (1:500, Thermo Scientific, MN1040), anti-Human PHF-tau(AT8) (1:500, Thermo Scientific, MN1020), anti-tau (phospho-Ser422) antibody(1:500, GeneTex, GTX86147), anti-tau (Tau5) (1:500, Millipore, MAB361), anti-GFAP (1:1000, Millipore, MAB3402), anti-Iba1(1:1000, Wako, 019-19741), and anti-β -actin (1:5000, Abcam, ab9485). The next day, the blots were washed three times with TBS-T for 15 min and incubated in the specific secondary antibodies (1:5000, Thermo Fisher Scientific, 31462 and 31432) for 1 h at room temperature under constant agitation. After washing, the membrane was either probed with enhanced chemiluminescence (ECL) Western blotting substrate (Thermo Fisher Scientific, 34080) and with detection of luminescence (Tanon). Signal intensities were quantified using ImageJ and data analyzed using GraphPad.

### Crude synaptosomal fractions

Crude synaptosomal fractions from mouse and rat brains were prepared by homogenizing 100 mg tissue at 800 rpm in 1 mL lysis buffer (0.32 M sucrose, 5 mM Hepes, and 10 ml ddH_2_O containing one tablet of Complete Mini-Protease Inhibitor and 100 μL Phosphatase Inhibitor Cocktail 3, pH 7.5). This was followed by centrifugation at 1000 × *g* at 4 °C for 10 min, and the supernatant was transferred into 2-mL reaction tube and centrifuged at 12,000 × *g* at 4 °C for 20 min. Supernatant containing the light membrane fraction and soluble enzymes (S2) was directly frozen. The pellet containing crude synaptosomes (P2) was resuspended in 500 μl RIPA buffer and stored at −80 °C.

### Analysis of sarkosyl-insoluble and -soluble tau fractions

Brain cortex tissues from *App*^*NL-G-F*^ and control rats were extracted using either the sarkosyl or the RIPA method. For the sarkosyl extraction, tissues were homogenized in cold RIPA-A buffer without SDS (50 mM Tris-HCl, pH 7.6, 150 mM NaCl, 5 mM EDTA, pH 8.0, 1% Nonidet-P40, 0.5% Sodium deoxycholate, 50 mM Na-fluoride, 1 tablet of COMPLETE inhibitors and 10 μL 1000 × NaVO_4_). The homogenates were centrifuged at 20,000 × *g* for 20 min at 4 °C, and the supernatants (~2.5 mL) were transferred to 15 mL Falcon tube and add 3.0 mL RIPA-B (RIPA-A buffer with 1% N-lauroylsarcosine (Sarkosyl)). After 1 h incubation with rocking at room temperature, the homogenates were centrifuged for 70 min at 100,000 × *g* and the supernatants were collected as sarkosyl soluble fractions. To prepare the sarkosyl-insoluble fraction, the pellets were resuspended in RIPA buffer with SDS. After a brief sonication (2× pulses for 10 s at 40% intensity) to dissolve fibrils, the supernatants were collected. Protein concentrations were measured using the BCA kit, and the samples were analyzed with 10% SDS-PAGE gels.

### Histology

For the histological staining, the rats were anesthetized with Nembutal (pentobarbital sodium, 0.2 mL/100 g body weight), and sacrificed by decapitation at different age; brains were removed and weighed. Brains were fixed by submerging into 4% paraformaldehyde (PFA) in PBS, embedded into the paraffin, sectioned in corona plane and processed. For histological analyses, Paraffin-embedded brains were coronally sectioned at 5-μm thickness on a microtome, and mounted on APS-coated slides. For 5-μm thickness sections, they were deparaffinized, and stained with Hematoxylin and Eosin (Merck).

### Electron microscopy (EM)

The EM sample preparation for brain tissues (hippocampus, prefrontal cortex and entorhinal cortex) was performed as a previous report.^103^ Briefly, the rats were anesthetized with avertin (300 mg/kg, ip, Sigma) and perfused transcardially with 0.1 M phosphate buffer (PB, pH 7.4), followed by 4% PFA (Sigma) and 2.5% glutaraldehyde (GA, Sigma) in 0.1 M phosphate buffer (PB, pH 7.4). The brains were removed from the skull and the post-fixed with 4% PFA and 2.5% GA at 4 °C overnight. On the next day, the 200 μm sections of the brains were cut though a vibratome (Leica) and the samples of hippocampus, prefrontal cortex and entorhinal cortex were collected. The samples were first post-fixed in 2% OsO_4_ (Ted Pella) in 0.1 M PB (pH 7.4) at room temperature for 90 min. Then the staining buffer was replaced by 2.5% ferrocyanide (Sigma) in 0.1 M PB (pH 7.4) at room temperature for another 90 min. After being washed 3 times with 0.1 M PB, the samples were incubated with filtered thiocarbohydrazide (TCH, Sigma) at 40 °C for 45 min, and then fixed by 2% unbuffered 2% OsO_4_ for another 90 min. After incubating with 1% uranyl acetate (Merck) aqueous solution at 4 °C overnight, the samples were incubated with a lead aspartate solution which was prepared by dissolving 0.033 g lead nitrate (Sigma) in 5 mL 0.03 M aspartic acid (Sigma, pH 5.0) at 50 °C for 120 min. Then, the samples were dehydrated through a graded ethanol series (50%, 70%, 80%, 90%, 100%, 10 min each) and then were dehydrated with pure acetone. Finally, the samples were embedded by epon-812 resin (SPI). Ultrathin sections (50 nm) were cut in serial sections with a diamond knife (Diatome) using the system of automated tape-collecting ultramicrotome (ATUM),^104^ and then imaged using a scanning electron microscopy (Zeiss Gemini 300) with a resolution of 3 nm/pixel and dwell time of 2 *µ*s. Image J software was applied to quantify the synapses of the hippocampus, prefrontal cortex and entorhinal cortex between WT and AD groups. 3D reconstructions of hippocampal synapses were conducted using Amira software.

### MRI

A total of 10 pairs of rats (10 wild type and 10 homozygous *App*^*NL-G-F*^ rats) were scanned for MRI. The animals were anaesthetized using the mixture of O_2_ and isoflurane (5% for induction, with maintenance concentrations varying from 0.75% to 2%, depending on the animal). In vi*vo* T2-weighted anatomical image of each animal was acquired on a Bruker Biospec 94/30 USR preclinical MRI scanner (Bruker, Ettlingen, Germany), using 2D TurboRARE (turbo rapid acquisition with relaxation enhancement) sequence with the following parameters: field of view (FOV) 38 × 38 mm^2^, matrix 256 × 256, slice thickness 0.48 mm, repetition time (TR) 7533 ms, echo time (TE) 33 ms, flip angle (FA) 90°, RARE factor 8, and number of averages (NEX) 2. The reconstructed images have the voxel size of 0.1484 × 0.1484 × 0.48 mm^3^.

The statistical parameter mapping software (SPM 12, Wellcome Department of Cognitive Neurology, London, UK) with in-house MatLab scripts was employed to perform voxel-based morphometry (VBM)^105^ for calculating the volume of ventricle system of each animal. A study-specific anatomical template was built and tissue classification was performed using the Diffeomorphic Anatomical Registration Through Exponentiated Lie algebra (DARTEL) pipeline.^106^ First, the T2-weighted images were registered to the SIGMA in vivo rat brain template,^107^ resulting in individual T2-weighted images in the standard space with an isotropic spatial resolution of 0.15 mm. The unified segmentation method^108^ were then used to segment these images into gray matter (GM), white matter (WM) and cerebrospinal fluid (CSF). The tissue segmentations were used to generate the final study-specific template and GM, WM and CSF probability maps using the DARTEL pipeline. The individual GM, WM, and CSF segmentation were then spatially normalized to the study-specific template. Modulated tissue maps were generated by using the Jacobian determinant of the deformation field obtained in the normalization process to encode the expansion or contraction of voxels. Therefore, the tissue volume of each voxel was preserved in the modulated images and the tissue volume of a given region of interest (ROI) can be estimated as the summation of the modulated voxel intensities multiplied by the voxel size. The modulated tissue maps were spatially smoothed for noise reduction using a Gaussian kernel with full width at half maximum of 0.3 mm. The ROIs of the ventricle system, including the bilateral lateral ventricles, the third and the fourth ventricles were drawn on the study-specific template. The volumes of these ROIs were then calculated on the CSF maps for each animal. Two-sample two-tailed Student’s *t*-test was performed for each of the ROI volumes to identify any group difference.

### Locomotion ability test

All behavioral tests were conducted using male rats of WT and homozygous *App*^*NL-G-F*^ genotype, at age of 4-month. Two tests were performed to assess locomotor activity of these rats.

The open field test consisted of an open, dimly illuminated circular arena with a diameter of 100 cm and a wall height of 50 cm. Rats were introduced from the center of the site and allowed to walk freely for a 10 min trial period. Horizontal locomotor activity (moving distance), time spent and number of entries in the central area were recorded by a computer-based system (Noldus Information Technologies, Wageningen, Netherlands).

Motor coordination of rats was evaluated by rotarod test (San Diego Instruments, San Diego, CA). The test included 3 training days with four 90 s trails per day and 1 testing day. During each trial of the training day, rats were placed on the cylinder, which accelerated from 5 rpm to 15 rpm in 15 s and maintained at this speed for another 75 s. The interval between trials was at least 15 min. On the fourth day, animals were tested for three trials with 15 min inter-trial interval. For each trial, the rotarod was accelerated from 5 rpm to 40 rpm in 300 s. The latency to fall of each rat was recorded.

### Morris water maze test

The Morris water maze experiments were conducted in a circular pool (150 cm diameter) with black and non-toxic ink water (23 ± 2 °C). The maze was equally divided into four logical quadrants, and a hidden platform (8 cm diameter, 2 cm beneath the water) was placed in a constant location of one quadrant (target quadrant, or quadrant I). Spatial cues were distributed around the pool. Animals were brought to the testing room 1 h before training session for environmental adaptation. The training session lasted for 4 consecutive days with four trials per day, and was followed by a probe test on the fifth day. In each trial of training session, rats were randomly released into the water from three quadrants (excluding the target quadrant) of the pool, and allowed to have 60 s for finding the hidden platform and 10 s to rest on the platform. In case that a rat did not find the platform within 60 s, it was guided to find and stayed on the platform for 10 s, and escape latency was recorded as 60 s. The escape latency and distance for the animals to reach the platform was automatically recorded by the digital tracking software EthoVision (Noldus Information Technologies, Wageningen, Netherlands). On the fifth day, the hidden platform was removed and a 60 s probe test for spatial memory recall was performed. The time spent and number of entries in each quadrant were recorded.

### Paired associates learning (PAL) task

PAL task is an episodic-like memory test for rats, which required sophisticated ability of spontaneous object recognition and visual discrimination.^68^ Rats were food restricted to maintain approximately 85% of their original weight, after that they were trained in touchscreen-equipped operant conditioning chambers (ABET II, Lafayette Instrument Company, IN, USA). Whisker software and an ABET II system were used for monitoring behavioral performance in PAL task.

The PAL protocol consisted of pretraining, training and testing stages, as outlined before.^109^ Briefly, rats were pretrained through five phases for instrumental operant conditioning before the training stage. In the first phase, rats were brought to the touchscreen chambers for 30 min with 10 reward pellets in the pellet receptacle to habituate the environment for 2 days. In the second phase, rats were trained to nose-poke a stimulus, which varied in shape and pattern and were displayed randomly in one of three screen windows, for reward. Three reward pellets were delivered by pairing with a tone for 1 s and lighting of pellet receptacle if the rat correctly touched the stimulus in 30 s. Otherwise, one pellet was delivered if the stimulus was not touched. After a 20 s inter-trial interval, a new trial began. The second phase continued until rats completed 100 trials in 60 min for 1 day. In the third phase, the rats were required to compulsively touch the stimulus to receive one reward pellet, and no new trial was automatically initiated compared with second phase if the rat did not touch the stimulus. Rats were only moved to next phase after successfully completing 100 trials in 60 min for 1 day. In the fourth phase, initiating a trial required rats to learn to nose-poke into the pellet receptacle, indicated by a light in the receptacle. When the rat poked its nose into the receptacle, the pellet receptacle played a tone for 0.2 s paired with light off and a stimulus displayed on the touchscreen. As rats received one pellet through touching the stimulus, the receptacle light would go on again after a 20-s interval. Once more, the criterion was 100 trials in 60 min for 1 day. In the fifth phase, rats were taught that a punish signal would occur if they touched the incorrect screen. Rats that touched blank screen were punished by an illumination of house lights and a 5 s time out. After a 20 s interval, the same stimulus was repeatedly presented in next trial until a correct response was made. The criterion for this phase was 100 trials completed in 60 min for 2 consecutive days, and percentage accuracy greater than 80%.

*Percentage accuracy* = *correct responses/(correct* + *incorrect responses)* × *100*.

All stimuli used in the 5 phases of pretraining were different from the following training and testing stages.

Next, in the training stage, rats were taught to understand the rules of association of stimulus and its correct location by correction trials. The three stimuli (the hollow circle-shape, the solid circle-shape and the cross-shape) were paired with corresponding locations of three touchscreens (left, center and right). For each trial, two identical stimuli were presented simultaneously in 2 of the 3 touchscreens and one stimulus would always be in the correct location (S+) and the other in the incorrect location (S−). Thus, there were six possible trial types (Fig. 7i). A nose-poke to the correct S+ (i.e., the hollow circle-shape in the left location, the solid circle-shape in the center location, and the cross-shape stimulus in the right location) resulted in delivery of a reward, whereas incorrect responses resulted in a 5 s time-out and a 20 s interval period, followed by correction trial whereby the trial was repeated until the rat made a correct choice. Rats were given 100 trials per day for 14 days. The percentage accuracy for each rat was calculated every day. Both WT and Homo rats had comparable accuracy rates in this training stage (Supplementary information, Fig. S11d).

After the training stage was completed, the rats were placed on a PAL testing stage to examine their capacities in visuospatial paired association. Unlike the training stage, there was no correction trial in this testing stage. The 6 possible trial types took turns to occur randomly in 100 trials per day, and the same trial type was never presented more than twice in a row. A trial was scored as correct if a nose-poke occurred at a right stimulus in the right location. During the course of the 30-day PAL tests, the percentage accuracy in 100 trials of each rat was calculated every day.

### Statistical analyses

To analyze the results of Western blot, signal intensities were quantified using ImageJ and analyzed with GraphPad Prism (version 8). For Immunostaining quantification including cell counting, at least three sections with the same stereo-location from at least three WT or *App*^*NL-G-F*^ rats were imaged on an Olympus FluoView FV1000 BX2 upright confocal microscope and images were quantified using Image J and Imaris8 softwares. Statistical differences were evaluated using unpaired *t*-test or one-way analysis of variance (ANOVA) followed by Boneferroni test with Graphpad Prism. All data were shown as means ± SEM.

For EM analyses, Image J software was applied to quantify changes in the ultrastructural elements of synapses of the hippocampus, prefrontal cortex and entorhinal cortex. 3D reconstruction of hippocampal synapses was conducted using Amira software. Kolmogorov-Smirnov test (non-parametric test) was used to compare cumulative distributions.

To analyze the results of MRI, the statistical parameter mapping software (SPM 12, Wellcome Department of Cognitive Neurology, London, UK) with in-house MatLab scripts was employed to perform voxel-based morphometry (VBM)^105^ for calculating the volume of ventricle system of each animal. A study-specific anatomical template was built and tissue classification was performed using the Diffeomorphic Anatomical Registration Through Exponentiated Lie algebra (DARTEL) pipeline.^106^ First, the T2-weighted images were registered to the SIGMA in vivo rat brain template,^107^ resulting in individual T2-weighted images in the standard space with an isotropic spatial resolution of 0.15 mm. The unified segmentation method^108^ was then used to segment these images into GM, WM and CSF. The tissue segmentations were used to generate the final study-specific template and GM, WM and CSF probability maps using the DARTEL pipeline. The individual GM, WM, and CSF segmentation were then spatially normalized to the study-specific template. Modulated tissue maps were generated by using the Jacobian determinant of the deformation field obtained in the normalization process to encode the expansion or contraction of voxels. Therefore, the tissue volume of each voxel was preserved in the modulated images and the tissue volume of a given ROI can be estimated as the summation of the modulated voxel intensities multiplied by the voxel size. The modulated tissue maps were spatially smoothed for noise reduction using a Gaussian kernel with full width at half maximum of 0.3 mm. The ROIs of the ventricle system, including the bilateral lateral ventricles, the third and the fourth ventricles were drawn on the study-specific template. The volumes of these ROIs were then calculated on the CSF maps for each animal. Two-sample two-tailed Student’s *t*-test was performed for each of the ROI volumes to identify any group difference using GraphPad Prism.

In behavior tests, unpaired student t-test was used in statistical analyses of locomotor activity tests, two-way RM ANOVA was used for spatial learning curve, and unpaired *t*-test for probe test in water maze test. For PAL test, two-way RM ANOVA with LSD post hoc was used performed with GraphPad Prism.

## Supplementary information

Supplementary information, Figure S1

Supplementary information, Figure S2

Supplementary information, Figure S3

Supplementary information, Figure S4

Supplementary information, Figure S5

Supplementary information, Figure S6

Supplementary information, Figure S7

Supplementary information, Figure S8

Supplementary information, Figure S9

Supplementary information, Figure S10

Supplementary information, Figure S11

Supplementary information, Figure S12

Video S1a 3D reconstruction of synapses in WT rat hippocampus

Video S1b 3D reconstruction of synapses in homozygous AppNL-G-F rat hippocampus

Video S2 3D reconstruction of ventricles in WT and homozygous AppNL-G-F rat brains
