# Supplementary material for: An App knock-in rat model for Alzheimer’s disease exhibiting Aβ and tau pathologies, neuronal death and cognitive impairments
Source: Cell Res. 2021 Nov 17;32(2):157–75. doi: 10.1038/s41422-021-00582-x (PMC8807612; doi:10.1038/s41422-021-00582-x)
Supplement: Supplementary file 1 — Supplementary information, Figure S1 [file 41422_2021_582_MOESM1_ESM.pdf]

Fig. S1

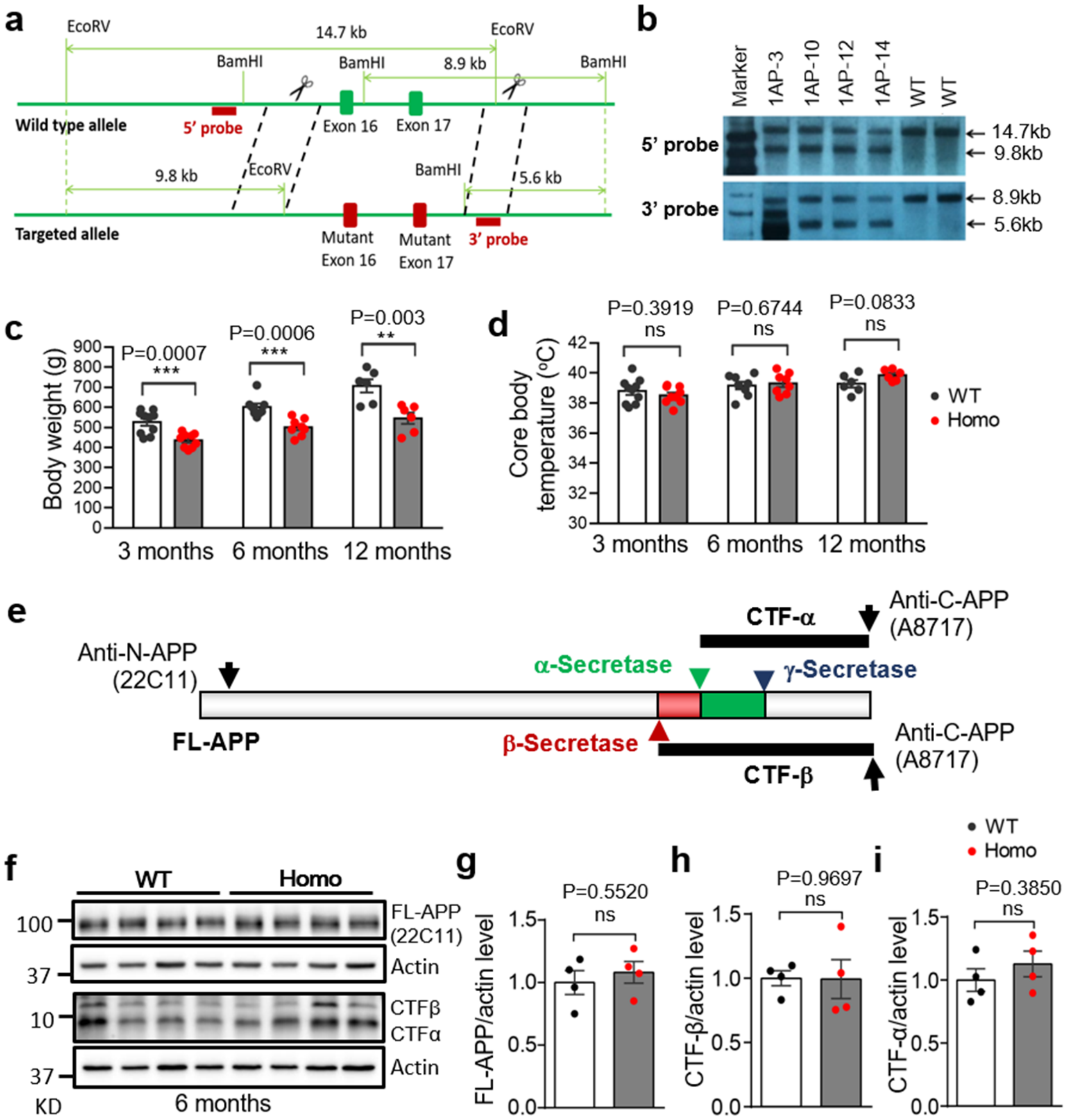
a
EcoRV
14.7 kb
EcoRV
BamHI
BamHI
8.9 kb
BamHI
Wild type allele
5' probe
Exon 16
Exon 17
9.8 kb
EcoRV
BamHI
5.6 kb
Targeted allele
Mutant Exon 16
Mutant Exon 17
3' probe
b
Marker
1AP-3
1AP-10
1AP-12
1AP-14
WT
WT
5' probe
14.7kb
9.8kb
3' probe
8.9kb
5.6kb
c
Body weight (g)
P=0.0007
***
P=0.0006
***
P=0.003
**
3 months
6 months
12 months
d
Core body temperature (°C)
P=0.3919
ns
P=0.6744
ns
P=0.0833
ns
3 months
6 months
12 months
WT
Homo
e
CTF-α
Anti-C-APP (A8717)
Anti-N-APP (22C11)
α-Secretase
γ-Secretase
FL-APP
β-Secretase
Anti-C-APP (A8717)
CTF-β
f
WT
Homo
100
FL-APP (22C11)
37
Actin
10
CTFβ
CTFα
37
Actin
KD
6 months
g
FL-APP/actin level
P=0.5520
ns
h
CTF-β/actin level
P=0.9697
ns
i
CTF-α/actin level
P=0.3850
ns
WT
Homo

Fig. S1. The generation of *App*$^{NL\text{-}G\text{-}F}$ rats and Aβ pathology in *App*$^{NL\text{-}G\text{-}F}$ rat brains. **a, b,** Southern blot analysis verifying the correct homologous recombination in F1 *App*$^{NL\text{-}G\text{-}F}$ rats. Diagram of cleavage sites by the restriction enzymes EcoRV and BamH1, as well as the locations of 5' Probe and 3' Probe, on the genomic DNA of WT and mutant alleles (**a**). Southern blot showing homologous recombination in F1 rats (**b**). Genomic DNA prepared from F1 rats were digested with EcoRV or BamH1, separated on agarose gel, and blotted with the 5' Probe and 3' Probe. The numbers shown on top of each lane are the ID numbers of F1 pups. For the WT allele, the 5' probe detected only one 14.7-kb band whereas the 3' probe detected one 8.9-kb band. For the heterozygous rats containing the targeted allele, the 5' probe 14.7 and 9.8-kb bands, whereas the 3' probe detected 8.9 and 5.6-kb bands, because one more EcoRV and BamH1 sites were inserted into the targeted allele. **c, d,** Body weight and core body temperature of *App*$^{NL\text{-}G\text{-}F}$ rats. Body weights (**c**) and core body temperatures (**d**) of 3-month-old, 6-month-old and 12-month-old wildtype (WT) and homozygous (Homo) *App*$^{NL\text{-}G\text{-}F}$ rats were examined and quantified as shown. **e-i,** Expression of full-length APP and its cleaved products. A schematic diagram showing the full-length APP and its cleaved products (**e**). Equal amounts of proteins from brain tissues of the homozygous *App*$^{NL\text{-}G\text{-}F}$ rats and their WT littermates (6 months of age) were separated on SDS-PAGE, and processed for Western blot, using specific antibodies as indicated. Densitometry was performed on each blot. A representative blot (**f**) is shown on the left and the quantifications (**g-i**) are shown on the right. n = 4 rats for each genotype. Data in this and bar graphs in all other figures are presented as mean ± s.e.m. Statistical analyses were carried out using t-test, P values are shown on top of the bar graphs.
