# Supplementary material for: An App knock-in rat model for Alzheimer’s disease exhibiting Aβ and tau pathologies, neuronal death and cognitive impairments
Source: Cell Res. 2021 Nov 17;32(2):157–75. doi: 10.1038/s41422-021-00582-x (PMC8807612; doi:10.1038/s41422-021-00582-x)
Supplement: Supplementary file 2 — Supplementary information, Figure S2 [file 41422_2021_582_MOESM2_ESM.pdf]

Fig. S2

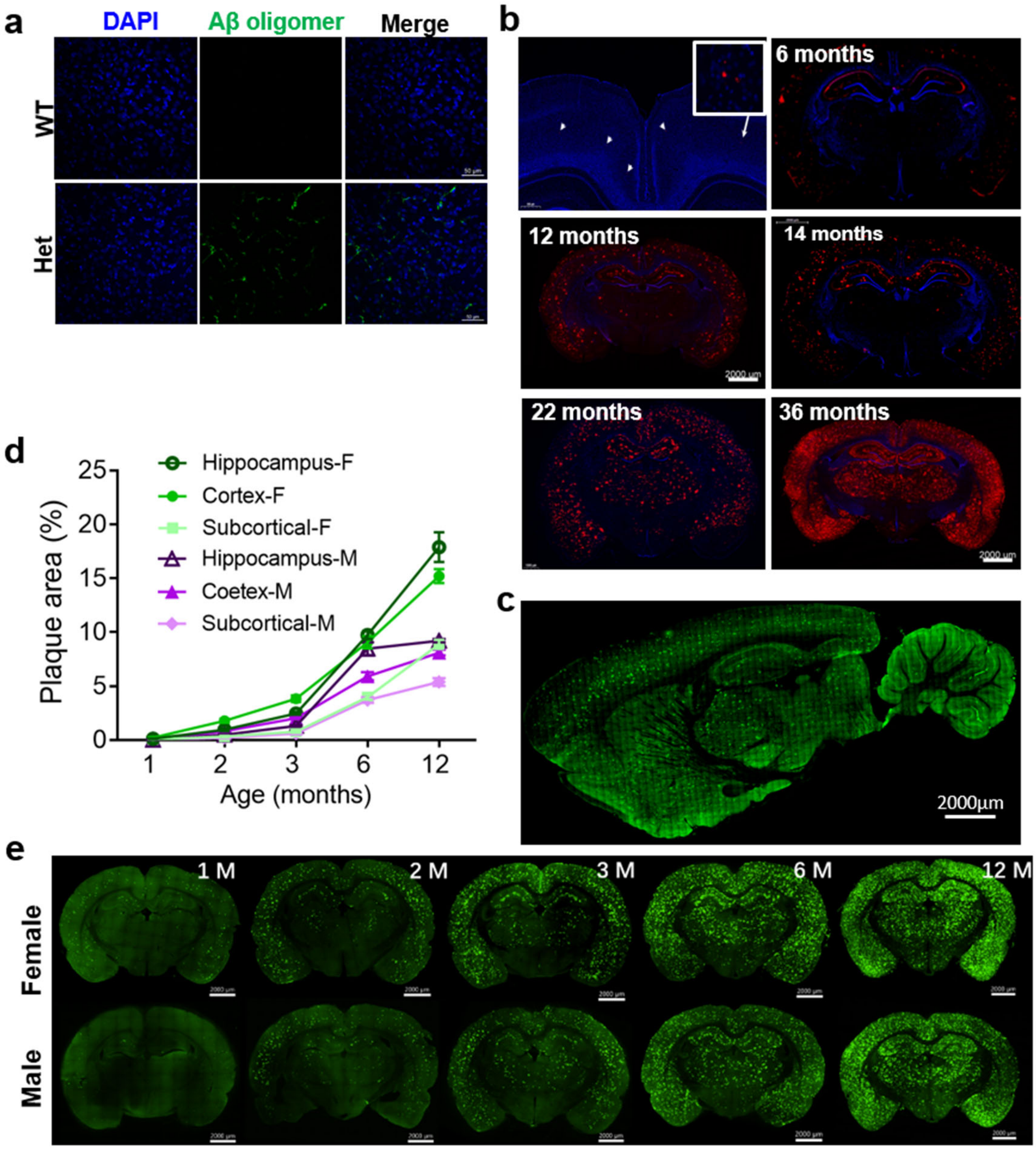
a
DAPI
Aβ oligomer
Merge
WT
Het
b
6 months
12 months
14 months
22 months
36 months
2000 μm
c
2000μm
d
Plaque area (%)
Age (months)
Hippocampus-F
Cortex-F
Subcortical-F
Hippocampus-M
Coetex-M
Subcortical-M
0
5
10
15
20
25
1
2
3
6
12
e
1 M
2 M
3 M
6 M
12 M
Female
Male

Fig. S2. Aβ pathology in *App*$^{NL\text{-}G\text{-}F}$ rat brains.

a, Expression of Aβ oligomers in Heterozygous (Hetero) *App*$^{NL\text{-}G\text{-}F}$ rats. The experiments were performed exactly the same as in (**Fig. 1d**) except 3-month-old WT and Hetero rat brain sections were used. Aβ oligomers were detected by an Aβ oligomer-specific monoclonal antibody (OMAB). n=4 pairs of rats. **b,** A gradual increase in Aβ deposition with age in heterozygous *App*$^{NL\text{-}G\text{-}F}$ brains. Brain sections from different ages (as indicated) of heterozygous *App*$^{NL\text{-}G\text{-}F}$ rats were stained with the anti-Aβ antibody (red) and DAPI-staining for nuclei (blue). Images were captured with the Light-sheet fluorescent microscope. Arrows indicate representative plaques. Scale bars represent 2000 μm. **c,** Representative microphotographs showing the lack of Aβ deposition in the cerebellum of *App*$^{NL\text{-}G\text{-}F}$ rat. Cerebellum sections from 10-month-old homozygous *App*$^{NL\text{-}G\text{-}F}$ rats were stained with an antibody specific for Aβ plaques and images were captured with the high-speed confocal microscope (Andor Dragonfly). Scale bars represent 2000 μm. **d, e,** Aβ deposition in female *App*$^{NL\text{-}G\text{-}F}$ rat brains. Brain sections from different ages of female and male homozygous *App*$^{NL\text{-}G\text{-}F}$ rats were stained with an antibody specific for Aβ plaques and images were captured with a light-sheet fluorescence microscope (**e**). Quantification of the plaque areas for different brain regions is shown in (**d**). F: female; M: male. n =5-6 rats for each time point, Scale bars: 2000 μm.
