# Supplementary material for: An App knock-in rat model for Alzheimer’s disease exhibiting Aβ and tau pathologies, neuronal death and cognitive impairments
Source: Cell Res. 2021 Nov 17;32(2):157–75. doi: 10.1038/s41422-021-00582-x (PMC8807612; doi:10.1038/s41422-021-00582-x)
Supplement: Supplementary file 3 — Supplementary information, Figure S3 [file 41422_2021_582_MOESM3_ESM.pdf]

Fig. S3

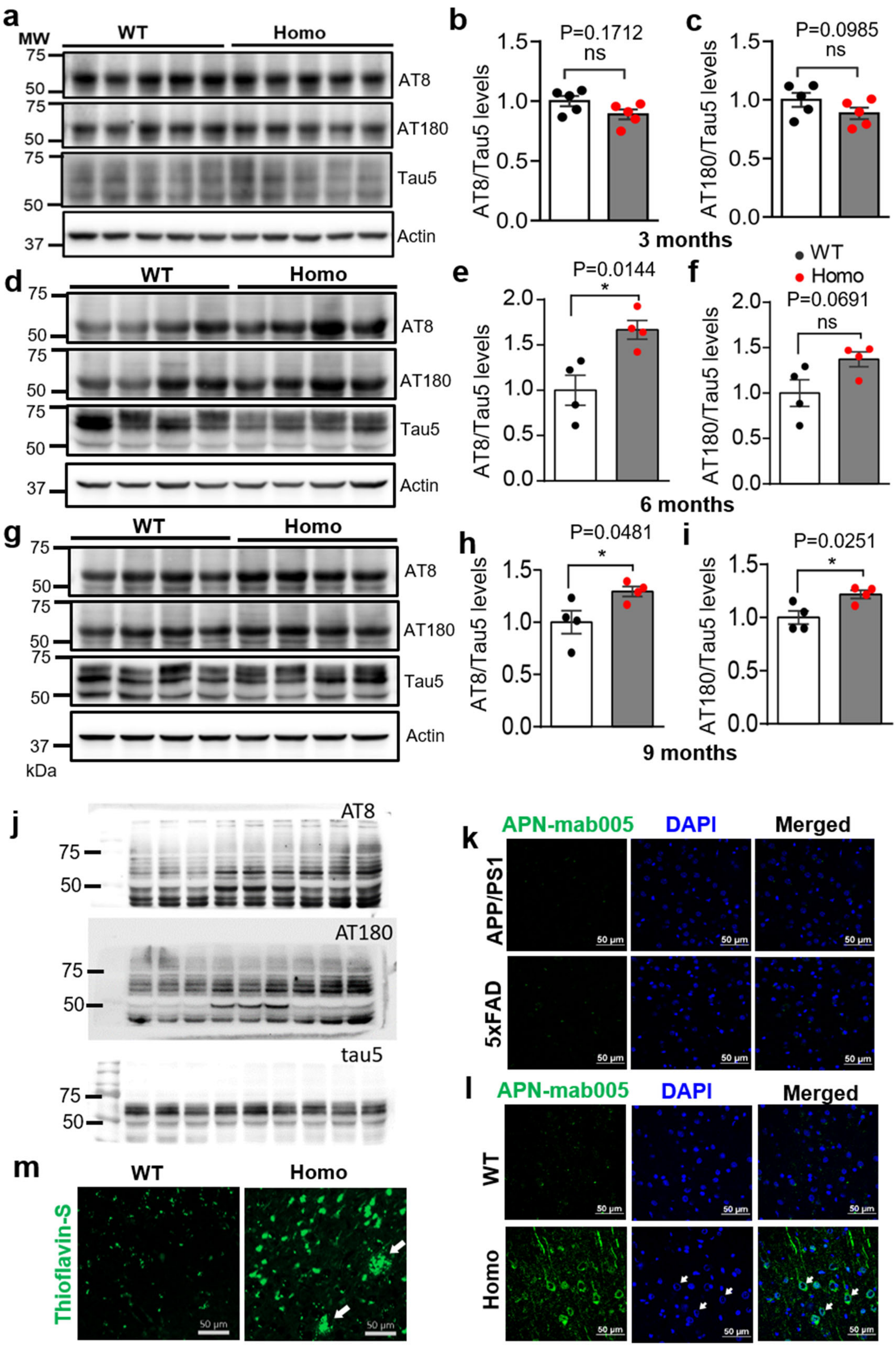

a
MW
WT
Homo
75
50
AT8
AT180
Tau5
Actin
37
b
P=0.1712
ns
AT8/Tau5 levels
1.5
1.0
0.5
0.0
c
P=0.0985
ns
AT180/Tau5 levels
3 months
d
e
P=0.0144
*
2.0
f
WT
Homo
P=0.0691
6 months
g
h
P=0.0481
i
P=0.0251
9 months
kDa
j
AT8
AT180
tau5
k
APN-mab005
DAPI
Merged
APP/PS1
5xFAD
50 μm
l
WT
Homo
m
Thioflavin-S

Fig. S3. Tau pathology in *App*$^{NL\text{-}G\text{-}F}$ rat brains.

a-i, Tau phosphorylation in 3-month (**a-c**), 6-month (**d-f**) and 9-month (**g-i**) old homozygous *App*$^{NL\text{-}G\text{-}F}$ rats. RIPA fractions of cortical lysates were immunoblotted with antibodies recognizing phosphorylated tau at residue S202/T205 (AT8), or T231 (AT180), and total tau protein was also detected as the control. The upper and lower bands in the AT8 and AT180 blots represent different tau isoforms. The levels of tau phosphorylation, quantified by densitometry and expressed as AT8/Tau5 and AT180/Tau5, are shown in the right. n=4-5 animals. **j,** The whole blots of AT8 and AT180 for **Fig. 2a.** **k,** Detection of tau aggregation using APN-mab005, a monoclonal antibody that recognizes only aggregated tau, mostly tau species segregated into the synaptic/membrane compartments. Frozen brain sections from 12-month old male APP/PS1 and 5xFAD mice were double stained with APNmab005, an antibody specifically aggregated tau (green), and DAPI in cortical region. Scale: 50 μm. **l,** Detection of tau aggregates in 22-month old homozygous *App*$^{NL\text{-}G\text{-}F}$ rats using APN-mab005. Frozen brain sections were double stained with APNmab005 and DAPI in cortical region. Scale: 50 μm. **m,** Detection of tau pathology using thioflavin-S staining. Brain sections (paraffin) from 22-month old male homozygous *App*$^{NL\text{-}G\text{-}F}$ rats were stained with thioflavin-S (green). White arrows indicate some Aβ plaques, which can also be detected by thioflavin-S staining. Scale: 50 μm.
