# Supplementary material for: An App knock-in rat model for Alzheimer’s disease exhibiting Aβ and tau pathologies, neuronal death and cognitive impairments
Source: Cell Res. 2021 Nov 17;32(2):157–75. doi: 10.1038/s41422-021-00582-x (PMC8807612; doi:10.1038/s41422-021-00582-x)
Supplement: Supplementary file 4 — Supplementary information, Figure S4 [file 41422_2021_582_MOESM4_ESM.pdf]

Fig. S4

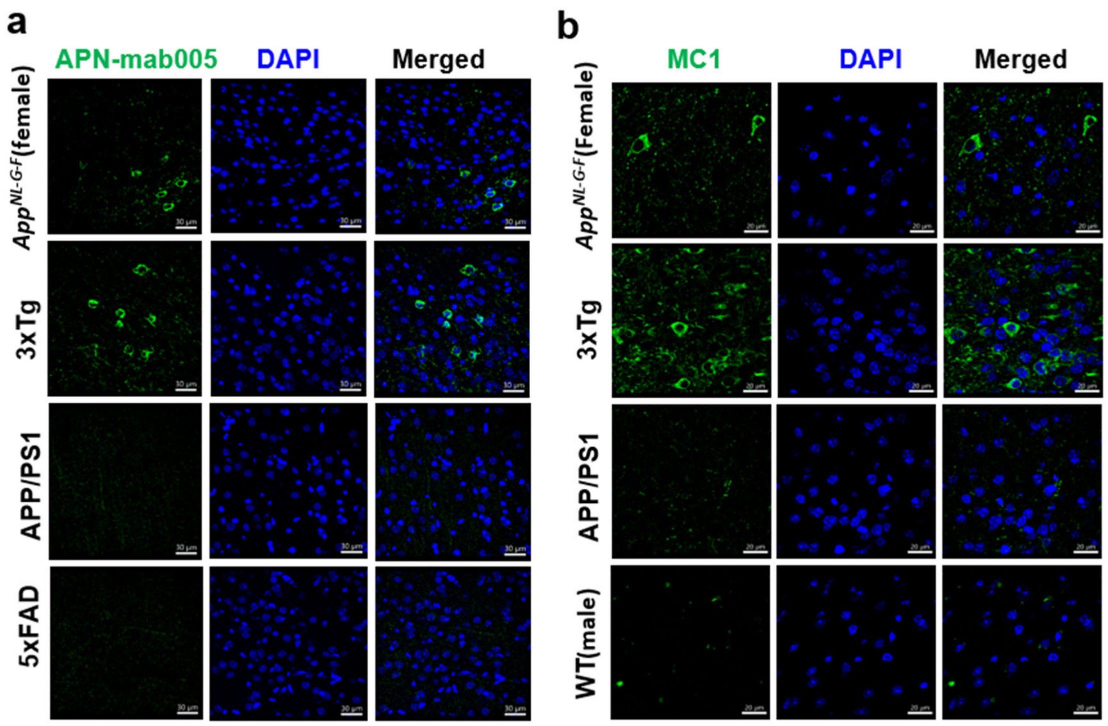

Fig. S4. Tau pathology in female *App*$^{NL\text{-}G\text{-}F}$ rat brains.

a, Detection of tau aggregates using APN-mab005. Frozen brain sections from 12-month-old female homozygous *App*$^{NL\text{-}G\text{-}F}$ rats were double stained with APNmab005 (green) and DAPI in cortical region. The 3xTg-AD mice section was used as a postive control. The APP/PS1 and 5XFAD sections were used as negative controls. Scale: 50 μm. **b,** Detection of disease-specific conformational change of tau using MC1 antibody. Brain sections from 12-month-old female homozygous *App*$^{NL\text{-}G\text{-}F}$ rats were double stained with the MC1 antibody (green) and DAPI in cortical region. The 3xTg-AD mice section was used as a postive control. The APP/PS1 and male WT sections were used as negative controls. Scale: 20 μm.
