# Supplementary material for: An App knock-in rat model for Alzheimer’s disease exhibiting Aβ and tau pathologies, neuronal death and cognitive impairments
Source: Cell Res. 2021 Nov 17;32(2):157–75. doi: 10.1038/s41422-021-00582-x (PMC8807612; doi:10.1038/s41422-021-00582-x)
Supplement: Supplementary file 5 — Supplementary information, Figure S5 [file 41422_2021_582_MOESM5_ESM.pdf]

Fig. S5

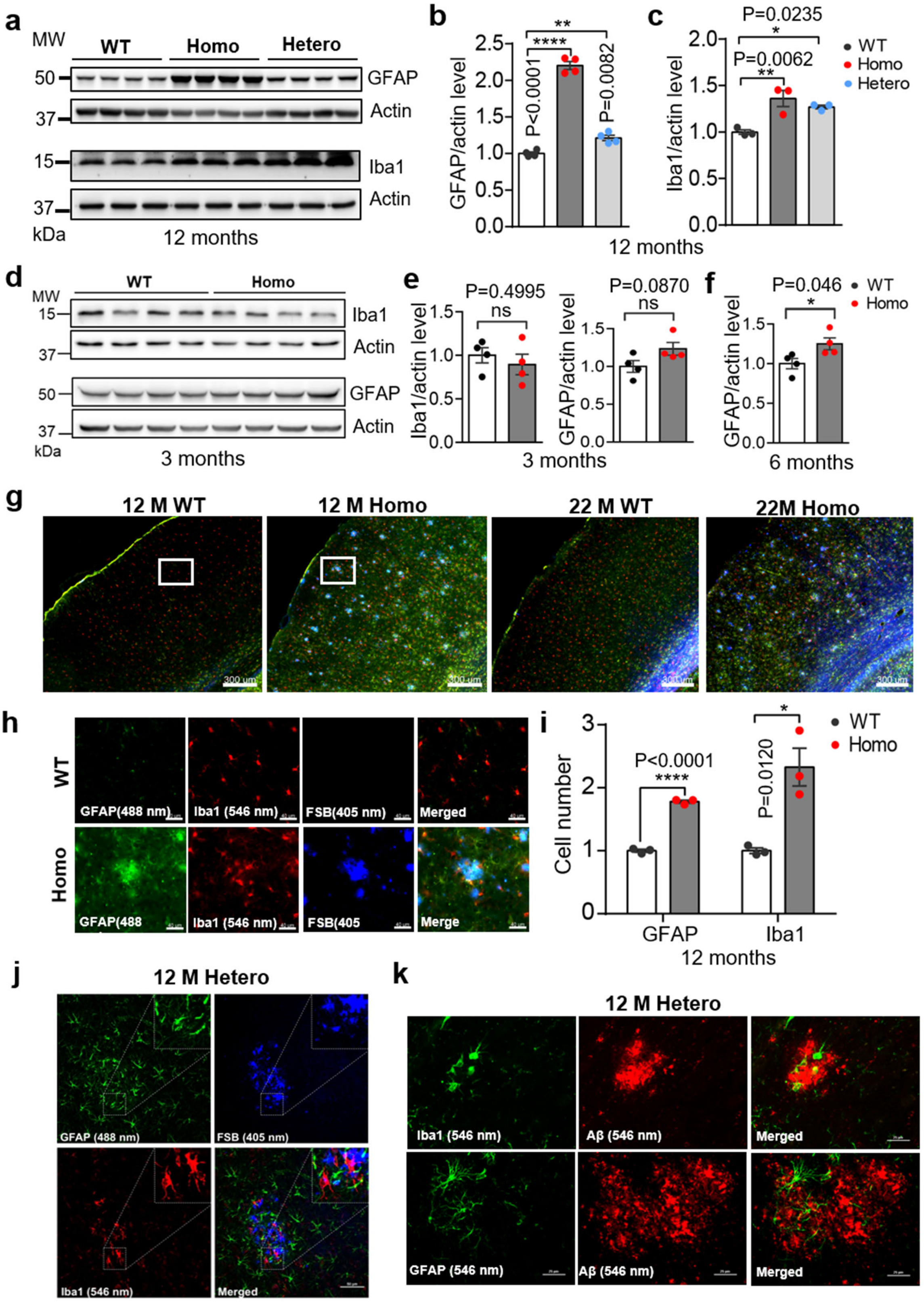
a
MW
WT
Homo
Hetero
50
GFAP
37
Actin
15
Iba1
37
Actin
kDa
12 months
b
GFAP/actin level
P<0.0001
****
**
P=0.0082
c
P=0.0235
*
P=0.0062
**
Iba1/actin level
WT
Homo
Hetero
12 months
d
MW
WT
Homo
15
Iba1
37
Actin
50
GFAP
37
Actin
kDa
3 months
e
P=0.4995
ns
Iba1/actin level
P=0.0870
ns
GFAP/actin level
3 months
f
P=0.046
*
WT
Homo
GFAP/actin level
6 months
g
12 M WT
12 M Homo
22 M WT
22M Homo
300 µm
h
WT
Homo
GFAP(488 nm)
Iba1 (546 nm)
FSB(405 nm)
Merged
GFAP(488
Iba1 (546 nm)
FSB(405
Merge
i
P<0.0001
****
*
P=0.0120
WT
Homo
Cell number
GFAP
Iba1
12 months
j
12 M Hetero
GFAP (488 nm)
FSB (405 nm)
Iba1 (546 nm)
Merged
k
12 M Hetero
Iba1 (546 nm)
Aβ (546 nm)
Merged
GFAP (546 nm)
Aβ (546 nm)
Merged

Fig. S5. Biochemical and morphological analysis of gliosis in aged *App*$^{NL\text{-}G\text{-}F}$ rat brains.

a-f, Microgliosis and astrocytosis in 12-month-old, 3-month-old and 6-month-old *App*$^{NL\text{-}G\text{-}F}$ rats. Astrocyte marker GFAP and microglia marker Iba1 were detected in cortical lysates from WT, homozygous (Homo) and heterozygous (Hetero) *App*$^{NL\text{-}G\text{-}F}$ rats of different ages using Western blotting. Representative immunoblots (**a, d**) and quantification bar graphs (**b, c and e, f**) are shown in the left and right panels respectively. n =4 (GFAP), n =3-4 (Iba1). Statistical analyses were carried out using one-way ANOVA or T-test. P values are shown in the bar graphs. **g-i,** Representative microphotographs of microgliosis and astrocytosis in homozygous *App*$^{NL\text{-}G\text{-}F}$ cortex. Gliosis were detected with triple staining of frozen sections from 12 and 22-month-old homozygous *App*$^{NL\text{-}G\text{-}F}$ rat, using fluorostyryl benzene (FSB, for Aβ plaque, blue), anti-GFAP (astrocytes, green) antibody and anti-Iba1 (microglia, red) antibody (**g**). The boxed areas in (**g**) are shown in the panels below at a higher magnification (**h**). Note that microglia and astrocytes are more concentrated around Aβ plaques. Scale bars represent 40 μm. The numbers of astrocytes and microglia are quantified (**i**). n=3. Scale bars represent 300 μm (**g**) and 40 μm (**h**) respectively. **j, k,** Microgliosis and astrocytosis in heterozygous *App*$^{NL\text{-}G\text{-}F}$ rats. Inflammatory responses were detected by staining using astrocytes (GFAP), Aβ plaques (FSB (**j**) and Aβ antibody (**k**)), and microglia (Iba1) in heterozygous *App*$^{NL\text{-}G\text{-}F}$ rat brains. Scale bars represent 50 μm (**j**) and 25 μm (**k**) respectively.
