# Supplementary material for: An App knock-in rat model for Alzheimer’s disease exhibiting Aβ and tau pathologies, neuronal death and cognitive impairments
Source: Cell Res. 2021 Nov 17;32(2):157–75. doi: 10.1038/s41422-021-00582-x (PMC8807612; doi:10.1038/s41422-021-00582-x)
Supplement: Supplementary file 6 — Supplementary information, Figure S6 [file 41422_2021_582_MOESM6_ESM.pdf]

Fig. S6

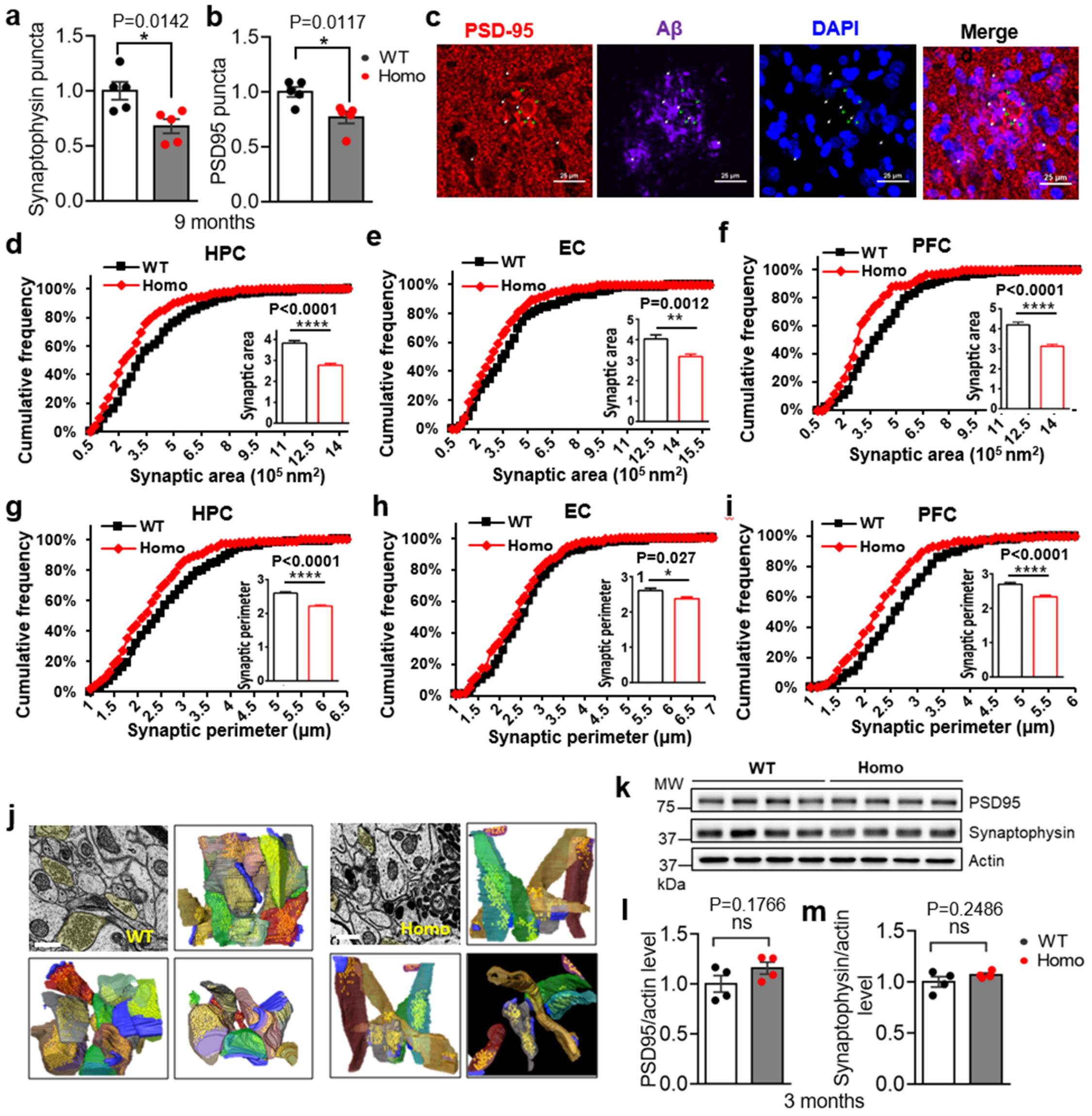

a
P=0.0142
Synaptophysin puncta
b
P=0.0117
PSD95 puncta
WT
Homo
9 months
c
PSD-95
Aβ
DAPI
Merge
d
HPC
e
EC
f
PFC
Cumulative frequency
Synaptic area (10⁵ nm²)
Synaptic area
P<0.0001
P=0.0012
g
HPC
h
EC
i
PFC
Synaptic perimeter (μm)
Synaptic perimeter
P<0.0001
P=0.027
j
WT
Homo
k
MW
PSD95
Synaptophysin
Actin
kDa
l
P=0.1766
ns
PSD95/actin level
m
P=0.2486
ns
Synaptophysin/actin level
3 months

Fig. S6. Enhanced Synaptic degeneration in *App^{NL-G-F}* rats.

a, b, Quantification of synapse density in the regions where no plaques in 9-month-old *App^{NL-G-F}* rats. The quantified images were derived from the experiments of **Fig. 4d**. **c,** Representative images of PSD95 immunostaining. Brain sections from 12-month-old homozygous *App^{NL-G-F}* rat were triple immunostained with fluorostyryl benzene (FSB, purple) for Aβ plaque, DAPI (blue), and PSD95 (red) antibodies for postsynaptic markers, respectively. White and green arrows respectively indicate swelling and hollowing of postsynaptic density, respectively. Scale bars represent 25 μm. **d-f,** Quantification of ***synapse area*** in hippocampus (**d**), entorhinal cortex (**e**) prefrontal cortex (**f**) from EM images of 6-month old WT and Homo rats by cumulative frequency plots. Insets display box plots of the actual synaptic area. At least 180 synapses were analyzed for each group. N = 3 rats / group. Statistics: Kolmogorov-Smirnov test (non-parametric test) was used to compare cumulative distributions. **g-i,** Quantification of ***synapse perimeter*** hippocampus (**g**), entorhinal cortex (**h**) prefrontal cortex (**i**) from EM images of 6-month old WT and Homo rats by cumulative frequency plots. Insets display box plots of the actual synaptic perimeter. At least 180 synapses were analyzed for each group. n = 3 rats/group. **j,** 3D reconstruction of synapses in hippocampus. Representative ATUM-SEM (Automated Tape-collecting UltraMicrotome - Scanning Electron Microscopy) image (upper left) and 3D reconstruction (other three) of synapses from WT (left) or homozygous *App^{NL-G-F}* (right) rats. Presynaptic terminals in pseudo colors (except blue) and post-synaptic densities in blue were reconstructed from a set of 50 ATUM-SEM image stacks (40 nm/slice). **k-m,** Levels of synaptic proteins in 3-month-old *App^{NL-G-F}* rats. Synaptosomal extracts from 3-month-old WT and Homo rat hippocampus were immunoblotted for presynaptic (synaptophysin) and postsynaptic (PSD95) markers (**k**). Quantifications of synaptic protein levels are shown in the right (**l, m**). n =4.
