# Supplementary material for: An App knock-in rat model for Alzheimer’s disease exhibiting Aβ and tau pathologies, neuronal death and cognitive impairments
Source: Cell Res. 2021 Nov 17;32(2):157–75. doi: 10.1038/s41422-021-00582-x (PMC8807612; doi:10.1038/s41422-021-00582-x)
Supplement: Supplementary file 7 — Supplementary information, Figure S7 [file 41422_2021_582_MOESM7_ESM.pdf]

Fig. S7

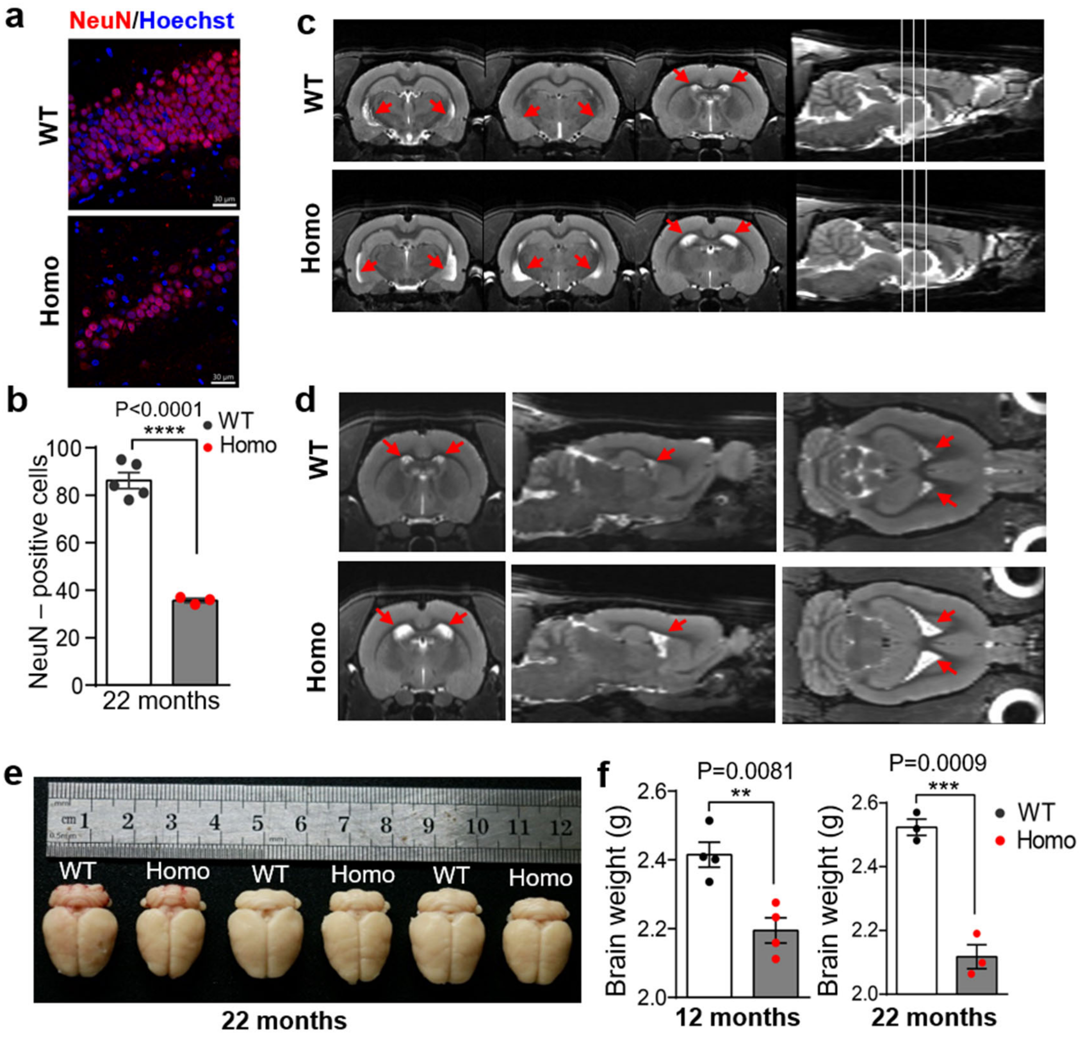

Fig. S7. Brain atrophy in *App*$^{NL\text{-}G\text{-}F}$ rats.

a,b, Neuronal loss in the hippocampus of 22-month-old *App*$^{NL\text{-}G\text{-}F}$ rats. Hippocampal CA1 sections from 22-month-old WT or homozygous (Homo) rat were stained with an antibody for NeuN and DAPI. Quantitations of NeuN-positive cells (red) in the stained sections are shown in (**b**). n =5 (Homo) or 3 (WT) rats. MRI images showing enlarged ventricles in 12-month-old WT and homozygous *App*$^{NL\text{-}G\text{-}F}$ (Homo) rats. **c,** In each row, three coronal slices are shown with slice thickness of 0.3 mm, and the rightmost sagittal image indicates the position of those slices. **d,** In each row, three orthogonal views of the slices going through the dorsal part of lateral ventricles are shown. The ventricles are indicated by red arrows. **e,** Representative brains of WT and Homo rats at 22 months of age. Note marked forebrain atrophy in the AD brain group. **f,** Plots of brain weights of WT and Homo rats at 12 and 22 months of ages. n=4 (12 months) or 3 (22 months).
