# Supplementary material for: An App knock-in rat model for Alzheimer’s disease exhibiting Aβ and tau pathologies, neuronal death and cognitive impairments
Source: Cell Res. 2021 Nov 17;32(2):157–75. doi: 10.1038/s41422-021-00582-x (PMC8807612; doi:10.1038/s41422-021-00582-x)
Supplement: Supplementary file 8 — Supplementary information, Figure S8 [file 41422_2021_582_MOESM8_ESM.pdf]

Fig. S8

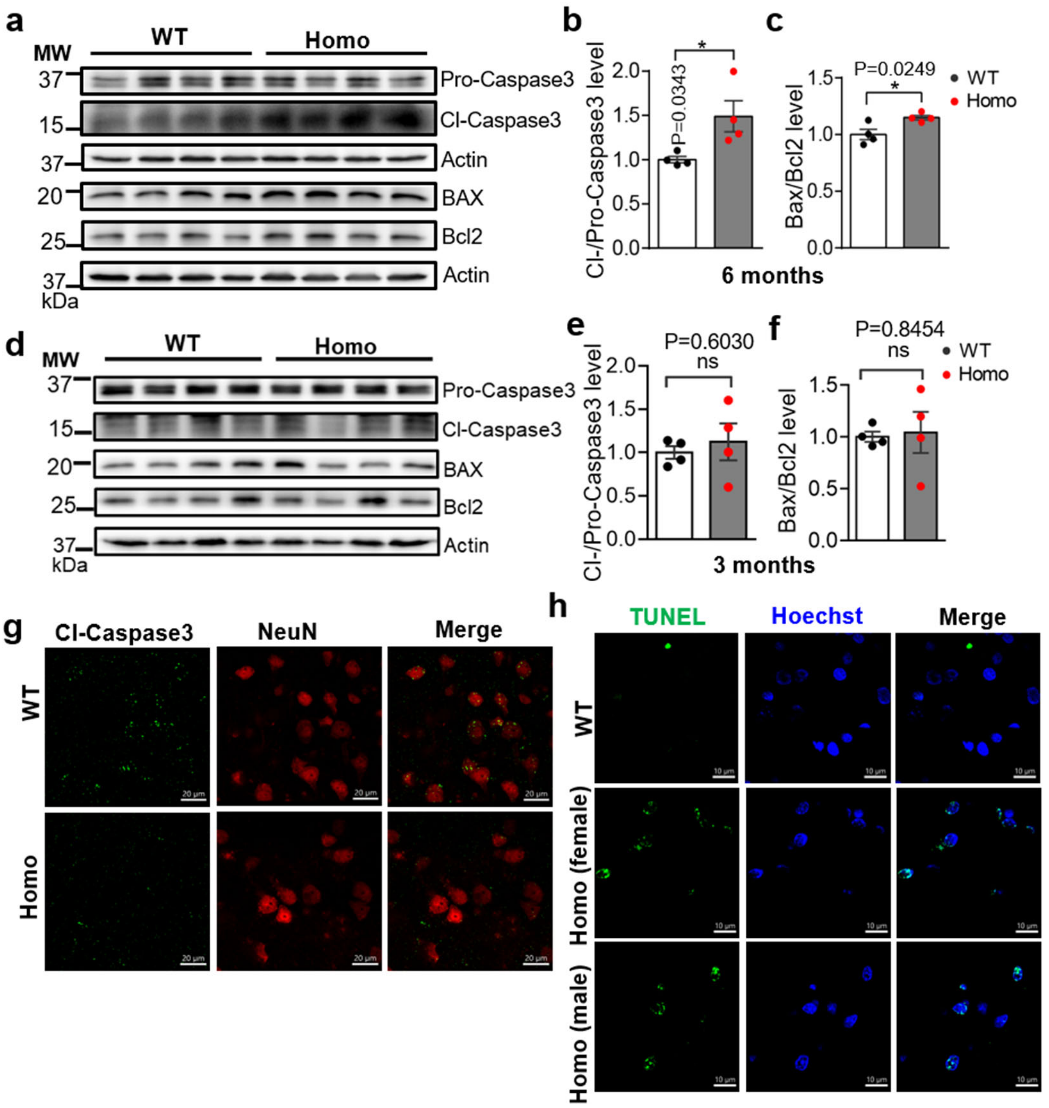

Fig. S8. Apoptosis in *App*$^{NL\text{-}G\text{-}F}$ rats.

a-f, Expression of apoptotic proteins in *App*$^{NL\text{-}G\text{-}F}$ rats. Hippocampal lysates from 6-month-old (**a**) and 3-month-old (**d**) WT, and homozygous *App*$^{NL\text{-}G\text{-}F}$ (Homo) rats were immunoblotted using anti-cleaved caspase3, anti-procaspase3, anti-Bax and anti Bcl-2 antibodies. Apoptosis was quantified by the ratios of cleaved caspase3 to procaspase3, as well as by the ratios of Bax to Bcl-2, shown on the right two panels (**b, c** and **e, f**). n = 4. **g,** Apoptosis in *App*$^{NL\text{-}G\text{-}F}$ rat neurons. Brain sections from 12-month-old WT and Homo rats were stained with anti-Cl-Caspase3 and NeuN antibodies and representative fluorescent images are shown. **h,** Apoptosis in female *App*$^{NL\text{-}G\text{-}F}$ rats. Brain sections from 12-month-old WT, female and male Homo rats were stained with TUNEL (green, apoptotic marker) and Hoechst (nucleus marker, blue) and representative fluorescent images are shown. Scale bars represent 20 μm.
