# Supplementary material for: An App knock-in rat model for Alzheimer’s disease exhibiting Aβ and tau pathologies, neuronal death and cognitive impairments
Source: Cell Res. 2021 Nov 17;32(2):157–75. doi: 10.1038/s41422-021-00582-x (PMC8807612; doi:10.1038/s41422-021-00582-x)
Supplement: Supplementary file 9 — Supplementary information, Figure S9 [file 41422_2021_582_MOESM9_ESM.pdf]

Fig. S9

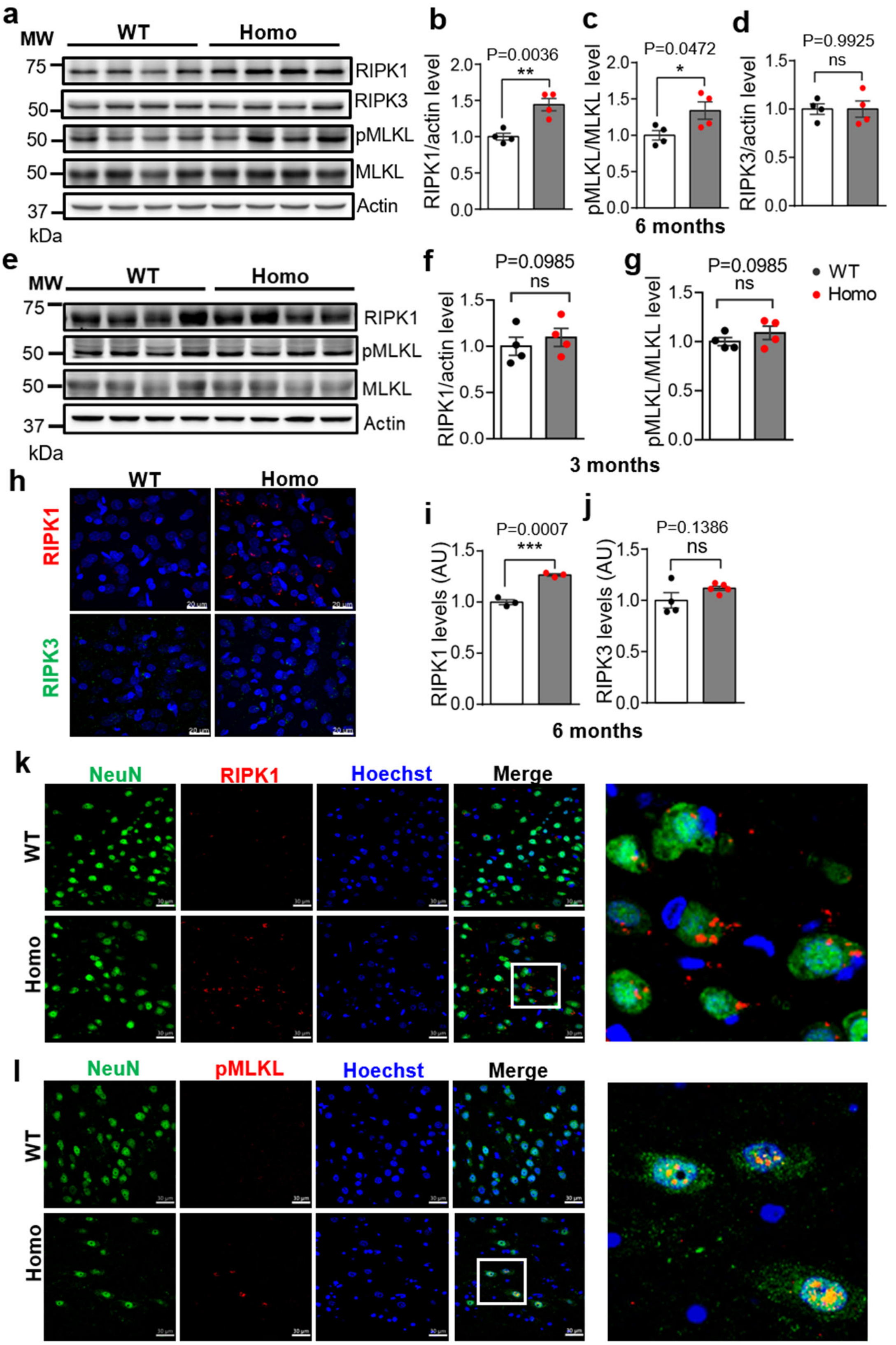

a
MW
WT
Homo
75
50
50
50
37
kDa
RIPK1
RIPK3
pMLKL
MLKL
Actin
b
P=0.0036
**
RIPK1/actin level
c
P=0.0472
*
pMLKL/MLKL level
d
P=0.9925
ns
RIPK3/actin level
6 months
e
f
P=0.0985
ns
RIPK1/actin level
g
P=0.0985
ns
pMLKL/MLKL level
WT
Homo
3 months
h
RIPK1
RIPK3
i
P=0.0007
***
RIPK1 levels (AU)
j
P=0.1386
ns
RIPK3 levels (AU)
6 months
k
NeuN
RIPK1
Hoechst
Merge
l
NeuN
pMLKL
Hoechst
Merge

Fig. S9. Necroptosis in *App*$^{NL\text{-}G\text{-}F}$ rats.

a-g, Expression of necrotic proteins in *App*$^{NL\text{-}G\text{-}F}$ rats. Hippocampal lysates from 6-month-old (**a**) and 3-month-old (**d**) WT, and homozygous *App*$^{NL\text{-}G\text{-}F}$ rats were immunoblotted using anti-RIPK1, anti-phosphorylated MLKL, anti-total MLKL, and anti-RIPK3 antibodies. The necroptosis levels were quantified by the ratio of RIPK1 to actin, the ratio of pMLKL to total MLKL, and the ratio of RIPK3 to actin (**b-d** and **f, g**). n = 4, Statistics: one-way ANOVA. **h-j,** Representative fluorescent images of brain sections from 6-month-old WT and Homo rats stained with anti-RIPK1 (red) and RIPK3 (green) antibodies (**h**). Scale bars represent 20 μm. Quantitative analyses of the immunoreactivity are shown in the right (**i, j**). n = 3. **k, l** Necroptosis in *App*$^{NL\text{-}G\text{-}F}$ rat neurons. Brain sections from 12-month-old WT and Homo rats were stained with NeuN and anti-RIPK1 (**k**) or anti-pMLKL (**l**) antibodies (red), and representative fluorescent images are shown. The boxed areas in the merged panels are shown in the right panels at a higher magnification level.
