# Supplementary material for: An App knock-in rat model for Alzheimer’s disease exhibiting Aβ and tau pathologies, neuronal death and cognitive impairments
Source: Cell Res. 2021 Nov 17;32(2):157–75. doi: 10.1038/s41422-021-00582-x (PMC8807612; doi:10.1038/s41422-021-00582-x)
Supplement: Supplementary file 10 — Supplementary information, Figure S10 [file 41422_2021_582_MOESM10_ESM.pdf]

Fig. S10

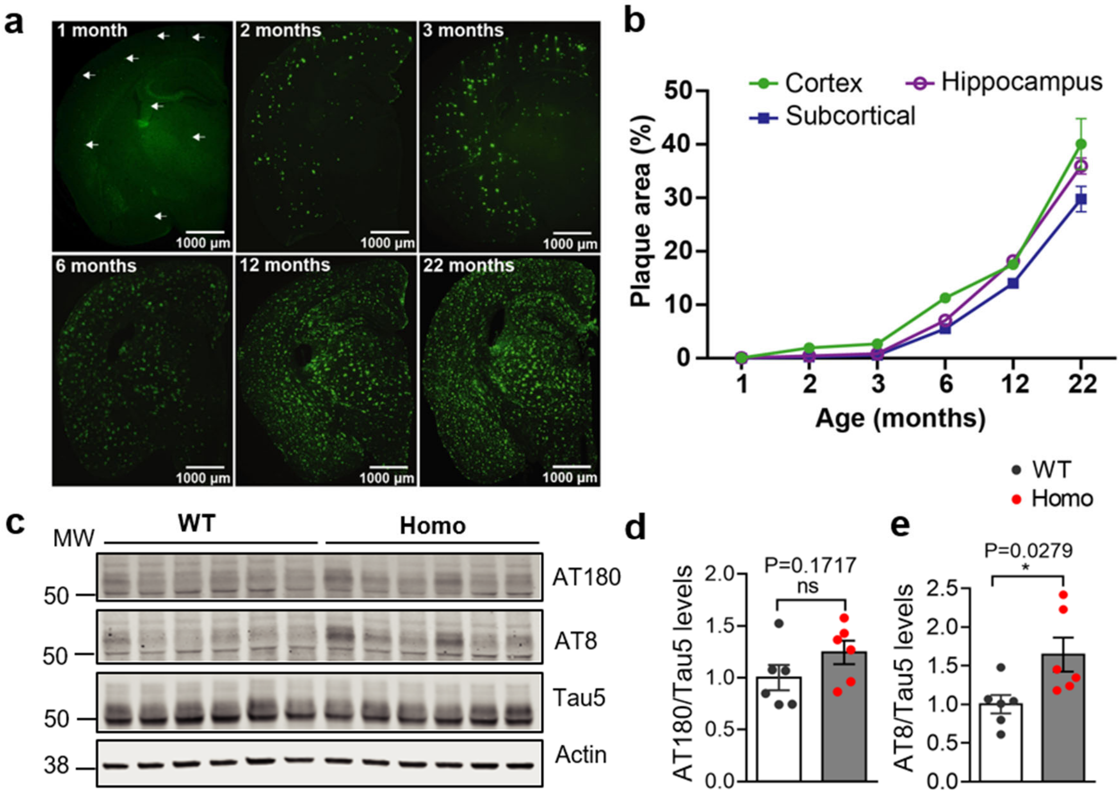

Fig. S10 Aβ pathology, tau phosphorylation in *App*$^{NL\text{-}G\text{-}F}$ mice.

a, Aβ deposition in *App*$^{NL\text{-}G\text{-}F}$ mouse brains. Brain sections from different ages of homozygous *App*$^{NL\text{-}G\text{-}F}$ mice were stained with an antibody specific for Aβ plaques and images were captured with a Nikon fluorescence microscope. Arrows in the "1-month" figure indicate representative plaques. Scale bars: 1000 μm. **b,** Quantification of the plaque areas for different brain regions is shown in (**a**). n =3 mice for each time point. **c-e,** Tau phosphorylation examined in *App*$^{NL\text{-}G\text{-}F}$ mice. (**c**) Cortical lysates from 12-month-old WT and Homo mice were immunoblotted with antibodies against phosphorylated tau at residue S202/T205 (AT8), T231 (AT180). Total tau protein and actin were used as loading controls. The levels of tau phosphorylation, quantified by densitometry and expressed as AT8/Tau5 and AT180/Tau5, are shown in the right (**d, e**). No significant difference was found between the two genotypes.
