# Supplementary material for: An App knock-in rat model for Alzheimer’s disease exhibiting Aβ and tau pathologies, neuronal death and cognitive impairments
Source: Cell Res. 2021 Nov 17;32(2):157–75. doi: 10.1038/s41422-021-00582-x (PMC8807612; doi:10.1038/s41422-021-00582-x)
Supplement: Supplementary file 11 — Supplementary information, Figure S11 [file 41422_2021_582_MOESM11_ESM.pdf]

Fig. S11

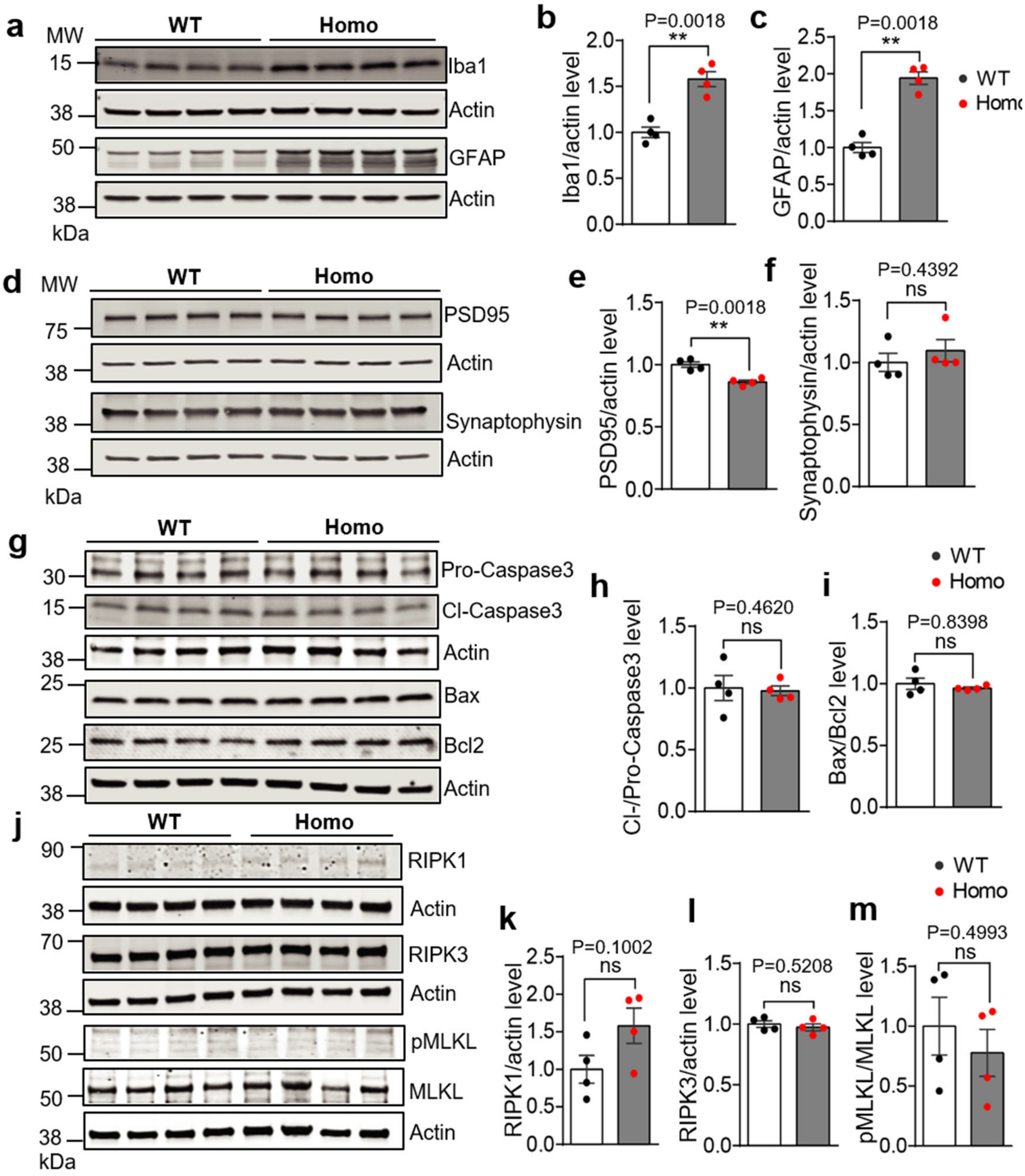

a
MW
WT
Homo
15
Iba1
38
Actin
50
GFAP
38
Actin
kDa
b
P=0.0018
**
Iba1/actin level
2.0
1.5
1.0
0.5
0.0
c
P=0.0018
**
GFAP/actin level
2.5
2.0
1.5
1.0
0.5
0.0
WT
Homo
d
MW
WT
Homo
PSD95
75
Actin
38
Synaptophysin
38
Actin
38
kDa
e
P=0.0018
**
PSD95/actin level
1.5
1.0
0.5
0.0
f
P=0.4392
ns
Synaptophysin/actin level
1.5
1.0
0.5
0.0
g
WT
Homo
Pro-Caspase3
30
Cl-Caspase3
15
Actin
38
25
Bax
Bcl2
25
Actin
38
h
P=0.4620
ns
Cl-/Pro-Caspase3 level
1.5
1.0
0.5
0.0
i
WT
Homo
P=0.8398
ns
Bax/Bcl2 level
1.5
1.0
0.5
0.0
j
WT
Homo
90
RIPK1
38
Actin
70
RIPK3
38
Actin
pMLKL
50
MLKL
50
Actin
38
kDa
k
P=0.1002
ns
RIPK1/actin level
2.5
2.0
1.5
1.0
0.5
0.0
l
P=0.5208
ns
RIPK3/actin level
1.5
1.0
0.5
0.0
m
WT
Homo
P=0.4993
ns
pMLKL/MLKL level
1.5
1.0
0.5
0.0

Fig. S11. Gliosis, synaptic alterations, and cell death in *App*$^{NL\text{-}G\text{-}F}$ mice.

a-c, Microgliosis and astrocytosis in *App*$^{NL\text{-}G\text{-}F}$ mice. Western blotting was used to detect microglia marker Iba1 and astrocyte marker GFAP in cortical lysates from 12-month-old WT and homozygous *App*$^{NL\text{-}G\text{-}F}$ (Homo) mice. Representative immunoblots and quantification bar graphs are shown in the left (**a**) and right (**b,c**) panels respectively. Protein lysates in this and the other 3 panels were derived from 4 Homo mice and 4 WT littermates (n = 4 pairs). Note that both microgliosis and astrocytosis were significantly increased in Homo mice. **d-f,** Synaptic alterations in *App*$^{NL\text{-}G\text{-}F}$ mice. Synaptosomal extracts from 12-month-old WT and Homo mouse hippocampus were immunoblotted for presynaptic (synaptophysin) and postsynaptic (PSD95) markers (**d**). Quantifications of synaptic protein levels are shown in the right (**e, f**). n =4. **g-i,** Lack of changes in apoptotic markers in *App*$^{NL\text{-}G\text{-}F}$ mice. (**g**) Hippocampal lysates from 12-month-old WT and Homo mice were immunoblotted using anti-cleaved caspase3, anti-procaspase3, anti-Bax and anti Bcl-2 antibodies. Apoptosis was quantified by the ratios of cleaved caspase3 to procaspase3, as well as by the ratios of Bax to Bcl-2, shown in the right two panels (**h, i**). No difference was found between the two genotypes. **j-m,** Expression of necroptotic markers in *App*$^{NL\text{-}G\text{-}F}$ mice. (**j**) Hippocampal lysates from 12-month-old WT and Homo mice were immunoblotted using antibodies against RIPK1, RIPK3, MLKL, phosphorylated MLKL. (**k-m**) The necroptosis levels were quantified by the ratios of RIPK1 to actin, RIPK3 to actin, the ratio of pMLKL to total MLKL. No difference was found between the two genotypes.
