# Supplementary material for: An App knock-in rat model for Alzheimer’s disease exhibiting Aβ and tau pathologies, neuronal death and cognitive impairments
Source: Cell Res. 2021 Nov 17;32(2):157–75. doi: 10.1038/s41422-021-00582-x (PMC8807612; doi:10.1038/s41422-021-00582-x)
Supplement: Supplementary file 12 — Supplementary information, Figure S12 [file 41422_2021_582_MOESM12_ESM.pdf]

Fig. S12

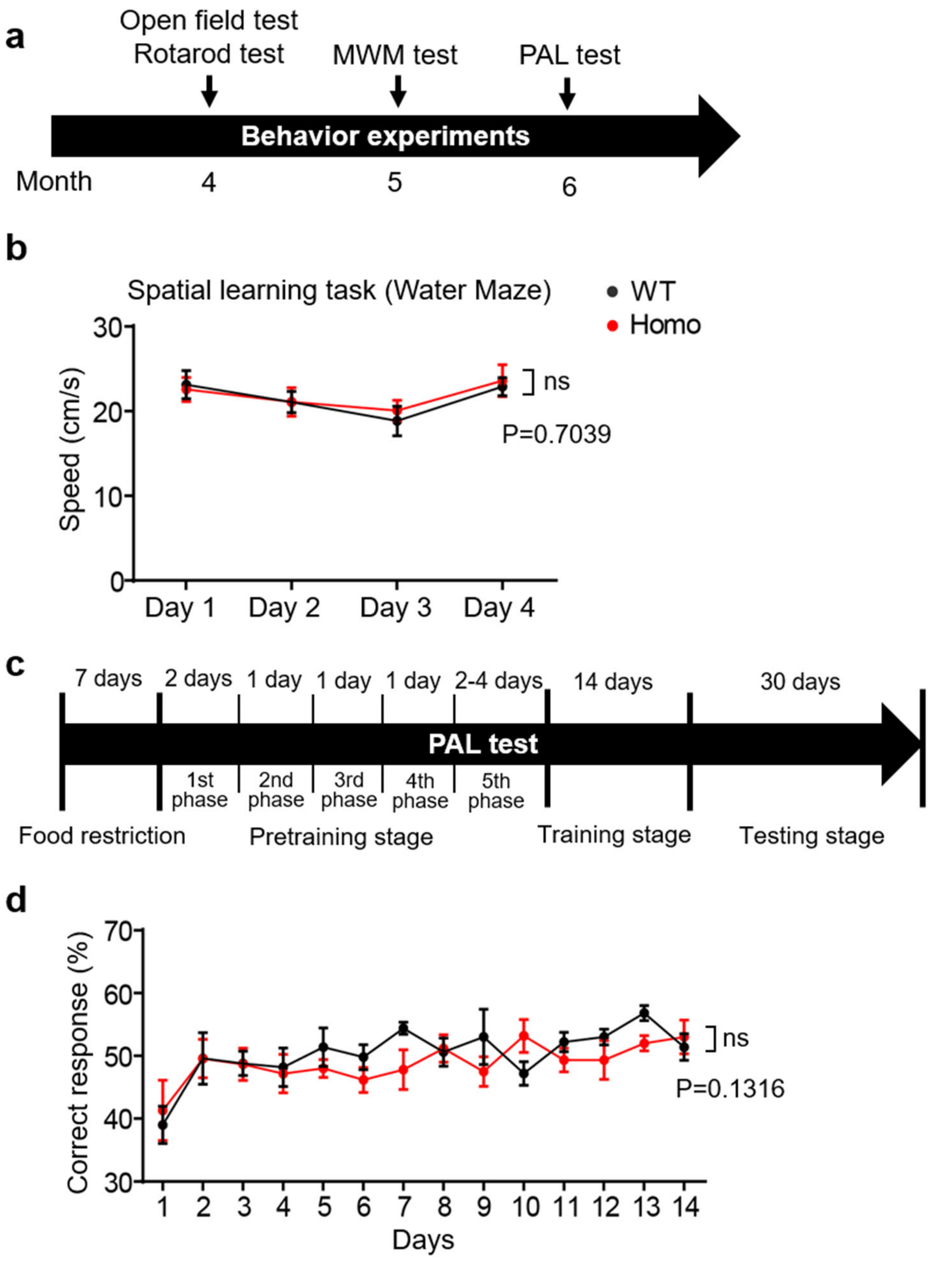

Fig. S12. Behavior analysis of WT and *App*$^{NL\text{-}G\text{-}F}$ rats.

a, Schematic diagram showing the time points when the behavioral tests were performed. **b,** Swimming speeds in Spatial learning of Morris Water Maze test in both experimental groups. WT and homozygous *App*$^{NL\text{-}G\text{-}F}$ (Homo) rats were subjected to learning paradigm of water maze for 4 days with four trials per day. n=8-9 rats/group. **c,** Schematic diagram showing the timeline when pre-training, training, and test stages of PAL were performed. **d,** Percentage accuracy of training stage in PAL task. Percentage accuracy of trails were collected all 14 days for WT and Homo rats in the training stage. Data are presented as mean ± s.e.m. Statistical analyses were carried out using two-way RM ANOVA.
